# Nano-Enabled Solutions for Plant Abiotic Stress Tolerance and Soil Contaminant Remediation: A Review

**DOI:** 10.3390/plants15040535

**Published:** 2026-02-08

**Authors:** Abdul Salam, Ali Raza Khan, Muhammad Zeeshan, Muhammad Siddique Afridi, Liupeng Yang, Qun Zheng, Zaid Ulhassan, Ruifei Wang, Zhixiang Zhang, Chen Zhao

**Affiliations:** 1 State Key Laboratory of Green Pesticide, South China Agricultural University, Guangzhou 510642, China; abdul_pk@zju.edu.cn (A.S.);; 2 School of Environment and Safety Engineering, Jiangsu University, Zhenjiang 212013, China; 3 Department of Plant Pathology, Federal University of Lavras (UFLA), Lavras 37200-900, MG, Brazil; 4 School of Breeding and Multiplication (Sanya Institute of Breeding and Multiplication), Hainan University, Sanya 572000, China

**Keywords:** agricultural sustainability, nano-enabled agriculture, growth-stimulators, stress tolerance, remediation

## Abstract

Global agriculture and food security are under serious threat from abiotic stresses, including salinity, drought, heavy metals, and extreme temperatures. While industrial development has driven progress, it has also intensified environmental challenges and contributed to declining crop productivity. Tackling these issues demands innovative and sustainable solutions. Nanotechnology has emerged as an effective approach to enhance stress tolerance, improve nutrient use efficiency, and increase crop yield and quality. This review critically examines the expanding role of nanoparticles (NPs) in mitigating abiotic stresses and promoting sustainable agricultural systems. While several studies have investigated the use of NPs in stress mitigation, ongoing research continues to reveal novel mechanisms and applications, highlighting the untapped potential of nanotechnology in plant science. The review discusses the impact of abiotic stress on plant growth and physiology, followed by a detailed analysis of the mechanisms through which NPs confer stress tolerance. Particular attention is given to the interaction of NPs with phytohormones and other growth regulators, as well as their role in the remediation of contaminated soils. Furthermore, the review highlights the dual role of NPs in stress alleviation and environmental remediation, while also considering emerging concerns about their potential ecological and toxicological impacts. Emphasis is placed on the need for risk assessments and effective management strategies. The review also identifies key knowledge gaps and methodological limitations, offering recommendations to guide future research in this emerging field.

## 1. Introduction

Plants play a crucial role in human life by cleansing air and soil, contributing to the global carbon cycle, preventing soil erosion, and providing essential nutrients for survival [1]. It has been reported that the global population is expected to surge rapidly in the following decades, with an increase of around 2.1 billion people compared to the current population [2]. The imbalance between global population growth and resource availability is driving a significant increase in crop demand. More regrettably, the occurrence of natural disasters such as floods, hail, and droughts has presented a considerable risk to the stability of the worldwide crop supply chain [3,4,5]. The primary drivers of low crop quality and yield are abiotic stresses (e.g., heavy metal contamination, drought, salinity, flooding), biotic factors (e.g., pests, pathogens), and nutrient deficiencies [6,7,8]. Thus, maintaining a balance between supply and demand in agricultural development requires enhancing crop output while retaining its high quality [9].

The extensive use of conventional fertilizers and synthetic pesticides has significantly increased crop productivity; however, it is widely acknowledged that these approaches have not proven to be long-term sustainable. Soil contamination with heavy metals (HMs) is another primary concern, as they can pose significant risks to crops and human health on a global scale [10,11]. Heavy metal contamination can occur due to a range of factors, including mining, industrialization, overexploitation, spills, combustion of fossil fuels, conventional agricultural practices, deposition from the atmosphere, and the improper disposal of metal-rich waste into water bodies and agrarian areas [12,13]. Drought and salt stress also severely impact crop health by disrupting plant metabolism, ultimately reducing growth, yield, and overall productivity. To address these constraints and boost agricultural productivity without expanding land use, we must adopt innovative, eco-friendly approaches to enhance sustainability and minimize ecosystem harm.

Integrating nanotechnology into agriculture has emerged as a promising approach offering novel solutions to address challenges [14,15]. Nanoparticles (NPs) can interact with plant systems at the molecular level, influencing stress responses, growth, and development. They can provide essential nutrients, protect plants from diseases and pathogens, and help detect or monitor trace components in the soil by interacting with their signals [16]. Since the early years of the twenty-first century, it has advanced sustainable agriculture and decreased the adverse effects on food and crop productivity [17]. This review examines the current agricultural challenges in the context of climate change and the subsequent applications of NPs in agriculture, highlighting both benefits and challenges. Different kinds of NPs have been reported to be successfully applied in agricultural sustainability, including metal and metal oxide NPs (e.g., ZnO, TiO_2_, Fe_2_O_3_, CuO, Si NPs, CeO_2_), carbon-based NPs (nanocarbon, CNTs, graphene derivatives), silica-based NPs, and nanocomposites. Based on the literature published on the role of NPs in agriculture sustainability, NPs induced improvement in plant resistance to adverse environmental conditions by (1) improving the photosynthesis-associated attributes, (2) enhancing the plant growth, (3) increasing the antioxidant defense system of plants, and (4) reducing the oxidative stress indicators (H_2_O_2_, O_2_, and electrolyte leakage). This review highlights eco-friendly approaches, comprehensive risk assessment, and regulatory frameworks essential for the safe and sustainable implementation of nanotechnology in agriculture. Furthermore, it identifies key research gaps and experimental limitations in the current literature, providing direction for future studies to enhance food safety and crop productivity, particularly in the context of global warming and increasing climate instability.

## 2. Agricultural Challenges and Food Insecurity

Agricultural challenges contribute to global food insecurity, affecting over one billion people through hunger, undernutrition, and malnutrition [18]. It is projected that severe hunger will continue to grow in Sub-Saharan Africa. In 2020, around 66.2% of the population had varying degrees of food insecurity, with 30% reporting severe and nearly 37% reporting moderate food insecurity [19]. According to 2021 estimates, 704 million people living in South Sahara face food insecurity [20]. The significance of agriculture in meeting the rising demands of a rapidly expanding population is indisputable [21].

**Figure 1:**
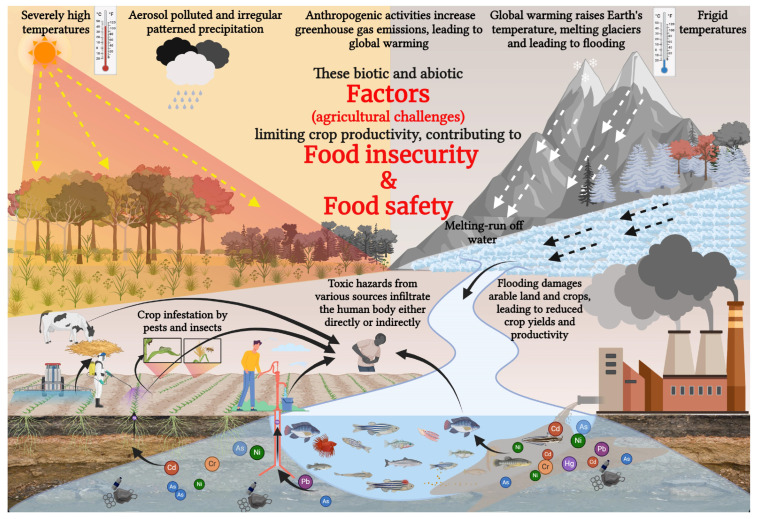
A schematic representation showing how abiotic factors cause environmental pollution and soil degradation, ultimately resulting in food insecurity and raising safety concerns. Anthropogenic activities cause a rise in global temperature, leading to global warming, melting glaciers, and rapid flooding, damaging arable soil. Severe temperature causes heat and drought stress, leading to irregular precipitation and limited crop productivity. HMs sourcing from various origins negatively affects crop production and poses direct or indirect risks to food safety and human health.

The leading causes contributing to agricultural problems and threats to food security include climate change, soil degradation, water shortages, HM pollution, plant diseases, and growing populations (Figure 1). These factors, individually or in combination, pose significant threats to the essential components of agriculture. The increase in global population intensifies the pressure on agriculture and food production, demanding enhanced crop productivity and sustainable agricultural practices. Integrating NPs into agrarian practices helps resolve present challenges and adheres to the ideals of environmental sustainability. It investigates the complex relationship between NPs and food security, providing insight into how the proper application of nanotechnology might contribute to a strong and sustainable global food system [15]. To understand the role of NPs in enhancing stress tolerance, it is essential first to examine how abiotic stresses affect plant growth and physiological processes.

## 3. Effect of Abiotic Stress on Plants

**Figure 2:**
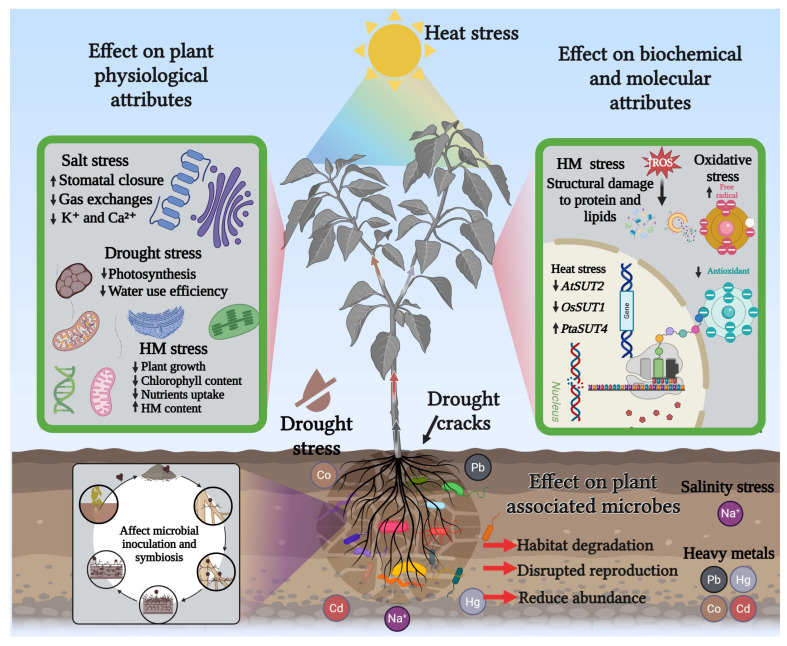
Impact of abiotic stress on plant physiological, biochemical, and molecular attributes and associated microbial ecology.

Abiotic stress refers to the adverse effects of non-living environmental factors on biological organisms. Extreme temperatures, salinity, drought, flooding, nutrient deficiency, and metal toxicity are major abiotic stressors that plants encounter throughout their life cycle. In agriculture, abiotic stress is a leading cause of substantial yield losses and economic damage [22]. Abiotic stresses such as drought, salinity, and HMs toxicity severely limit crop productivity and quality (Figure 2). These adverse conditions can impair plant growth and development, leading to yield reductions of up to 50–70% [23]. Plants are sessile and have developed complex signaling and response mechanisms to cope with these stressors, which involve dynamic changes at physiological, biochemical, and molecular levels [24]. Understanding these adaptive mechanisms is crucial for developing stress tolerant crop varieties and sustainable agricultural practices. Additionally, plant-associated microbes play a vital role in modulating stress responses and enhancing plant tolerance. However, the stressors mentioned above have a detrimental impact on these microbes, resulting in disturbed symbiotic niches and ultimately reducing plant growth and overall performance [25].

### 3.1. Effect of Abiotic Stress on Plant Physiological Attributes

Abiotic stress significantly alters essential physiological functions in plants, including photosynthesis, respiration, and development, thereby affecting plant growth and productivity [26]. When plants grow in soil contaminated with HM, they absorb these toxic metals, translocate them to different tissues, and accumulate them in cells, thereby disrupting vital physiological processes such as photosynthesis and ultimately compromising overall plant health [27]. For instance, HM stress has been reported to induce germination inhibition and early plant growth development [28]. Ulhassan et al. [29] reported a significant reduction in seed germination percentage in *Brassica napus* under chromium (Cr) exposure, indicating the high sensitivity of its seeds to Cr stress. Similarly, exposure to Cd at 2 μM has been shown to markedly reduce plant growth and chlorophyll content in rapeseed, primarily due to Cd accumulation in the roots and leaves [30]. Similarly, osmotic stress induced by drought or salinity leads to stomatal closure, which slows down plant growth by limiting gas exchange. This response also contributes to nutrient deficiencies, particularly of essential ions like K^+^ and Ca^2+^, and alters leaf photochemistry and carbon metabolism, thereby negatively affecting photosynthesis. As stomata close, the reduced availability of CO_2_ in the intercellular spaces further impairs the biochemical reactions of the Calvin cycle, diminishing the overall photosynthetic capacity [31]. Stomatal closure under heat stress is another major factor contributing to reduced photosynthesis due to limited CO_2_ diffusion. In addition, the decline in chlorophyll pigments under heat stress is often attributed to lipid peroxidation of chloroplast and thylakoid membranes, as observed in sorghum exposed to high temperatures (40/30 °C, day/night). Photosystem II efficiency, indicated by the Fv/Fm ratio, along with stomatal conductance, was also significantly reduced under these conditions. Collectively, these factors contributed to a notable decline in photosynthetic performance exposed to adverse conditions [32]. However, plants have evolved a variety of physiological responses to cope with abiotic stresses, which are essential for their survival under fluctuating environmental conditions. These responses can be further enhanced through innovative strategies and advanced technologies [33].

### 3.2. Effect of Abiotic Stress on Plant Molecular Attributes

Under abiotic stress, crops undergo a range of cellular, biochemical, and molecular alterations. These responses are regulated by complex signal transduction pathways that coordinate stress perception, signal relay, and activation of defense mechanisms, ultimately determining the plant’s tolerance or sensitivity. Upon exposure to stress, plants reprogram their defense networks and metabolic pathways to adapt and maintain cellular homeostasis [34]. Stress-specific signal transduction begins with the perception of environmental stress, followed by signal amplification through second messengers like Ca^2+^, NO, phospholipids, and protein kinases. Additionally, abiotic stress factors, such as drought, salinity, and HMs toxicity, lead to the overproduction of reactive oxygen species (ROS). Elevated ROS levels during stress conditions are harmful to plants, as highly reactive oxygen radicals damage essential cellular components, including lipids and proteins, thereby disturbing cellular water potential and leading to structural damage [34]. In particular, excessive ROS generated under abiotic stress overwhelm the antioxidant defense systems of plants, leading to oxidative damage of organelles and loss of cellular integrity. For example, chlorophyll is highly susceptible to ROS; excessive HM uptake can degrade its ultrastructure by promoting ROS accumulation and inhibiting its biosynthesis [35]. Furthermore, the accumulation of HMs in mitochondria disrupts the electron transport chain, resulting in electron leakage and the formation of superoxide radicals (O_2_•^−^). Both hydroxyl radicals (•OH) and superoxide radicals are highly reactive and capable of damaging critical organelles such as the nucleus, chloroplasts, and mitochondria [13]. The balance between ROS generation and elimination is crucial for plant acclimatization under abiotic stress. Failure to detoxify excess ROS can impair multiple cellular functions, reduce photosynthetic efficiency, and ultimately lead to decreased crop productivity. Similarly, extreme temperatures alter plant biochemical and physiological functions by modulating molecular mechanisms. For instance, heat stress down-regulates *AtSUT2* in Arabidopsis and *OsSUT1* in rice, affecting sucrose transport and grain quality, while up-regulating *PtaSUT4* in poplar, also linked to reduced sucrose translocation. Flooding stress induces molecular changes that support morphological and physiological adaptation. In Arabidopsis, five ethylene response factor (ERF) genes play key roles in flood and hypoxia tolerance. Among them, the ERF-VII transcription factor *SUB1A* is a critical regulator of submergence tolerance, with its expression activated only during flooding and rapidly diminishing once normal conditions resume.

### 3.3. Effect of Abiotic Stress on Plant-Associated Microbes

Plant-associated microbes are increasingly recognized for their potential to enhance plant growth in challenging environments. They employ various mechanisms to promote root development and improve water uptake [36]. Rhizospheric organisms exhibit a distinctive mode of existence, as multiple influencing factors shape their microbiome composition [37]. Among these, soil physicochemical properties play a pivotal role in driving changes in microbial communities, with HMs known to exert a significant impact on microbiome structure and diversity. For instance, disrupts the enzymatic activities of soil microorganisms by denaturing enzymes, damaging membrane structures, impairing cellular functions, and interfering with enzyme synthesis [38]. In addition, HMs hinder microbial reproduction and cause morphological and physiological abnormalities. Consequently, the presence of toxic HMs in the environment can adversely affect biodegradation processes. Moreover, abiotic stressors have detrimental effects on their habitat degradation (deforestation, land conversion, urbanization, etc.), and other soil contaminants such as antibiotics, plastics, pesticides, fertilizers, etc., that impose negative impacts on the soil microbiome [36]. The alteration of one factor can have either a positive or negative effect on others, ultimately leading to shifts in the structure of the soil microbiome. Similarly, salinity impairs plant growth and suppresses microbial activity in the soil due to elevated osmotic pressure, toxic ion accumulation, and nutrient imbalances. An increase in salinity triggers a rapid osmotic phase that slows down cell division and growth rates, creating a hostile environment for both roots and soil microorganisms [31]. Zhang et al. [39] reported that increasing soil salinity reduces the relative abundances of soil bacterial, fungal, and arbuscular mycorrhizal communities, thereby impairing their functional roles such as organic matter decomposition and lignin degradation in saline coastal ecosystems. Similarly, Santos-Medellín et al. [40] investigated the response of rice root-associated microbiomes to drought stress and observed significant compositional shifts in both rhizosphere and endosphere communities, particularly reflected in changes in the relative abundance of various taxonomically diverse bacterial groups.

## 4. Role of Nanoparticles in Improving Plant Growth and Stress Tolerance

Nano-enabled agriculture incorporates nanomaterials to enhance the efficacy and ecological sustainability of conventional farming practices. To date, various kinds of NPs have been successfully applied to agricultural sustainability. Based on structural composition, nanomaterials are broadly classified into four categories: organic, inorganic, carbon-based, and composite nanomaterials [41]. Organic nanomaterials, such as liposomes, micelles, dendrimers, and ferritin, are derived from organic compounds and are generally biodegradable, low-toxic, and well-suited for encapsulation due to their hollow structures, although they are sensitive to heat, light, and electromagnetic radiation [42]. Inorganic nanomaterials lack carbon and mainly include metal (e.g., Ag, Au) and metal oxide NPs (e.g., ZnO, TiO_2_) [43]. Carbon-based nanomaterials consist entirely of carbon and include carbon nanotubes, graphene, fullerenes, carbon nanofibers, and carbon black [41]. Composite nanomaterials are formed by combining NPs with other nanomaterials or bulk matrices, resulting in multifunctional properties [44]. When applied appropriately, nano-enabled practices can enhance agricultural productivity, improve food safety, and increase nutritional value while causing less harm to the environment than their traditional counterparts [2,45]. However, the advancement of nano-enabled agriculture also presents some challenges, including the requirement for appropriate safety standards and comprehending the mechanisms by which NPs infiltrate plants and their possible impact on the environment [46,47]. Those challenges are discussed in the corresponding section.

### 4.1. Nanoparticles as Plant Growth Stimulators

**Figure 3:**
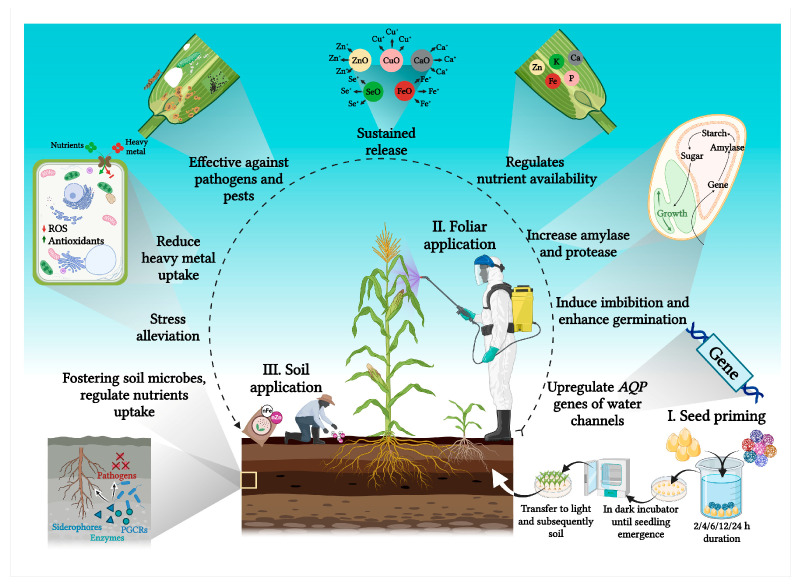
Illustration of NPs functioning as plant growth stimulators, highlighting their application methods and the complex modifications they induce in plants.

Nanoparticles as plant growth stimulators (PGSs) emerge as a promising avenue, offering potential solutions to conventional fertilization practices [48]. Studies have reported that NPs improve plant growth and development and reduce pollutants in the ecosystem, leading toward sustainable agriculture [49]. NPs offer notable benefits by enhancing plant–soil interactions and have been shown to positively influence plant growth and establishment (Table 1; Figure 3).

#### 4.1.1. Nutrient Delivery

Nutrients administered through NPs exhibit a gradual release spanning 40–50 days, contrasting with the 4–10 days observed in conventional bulk fertilizers [50]. Employing controlled delivery techniques ensures that the measured release of nutrients aligns with plant requirements over time. This controlled release mechanism reduces nutrient loss and adverse effects on soil, soil-beneficial microbiota, and plants. It addresses the challenge of continuously applying chemical fertilizers, often leading to waterway leaching [51,52]. When NPs are applied to soil, they tend to form aggregates, which reduces their effective surface area; as these aggregates increase in size, their mobility within porous media decreases [53]. However, the presence of organic matter, along with ambient conditions and the chemical properties of the NPs, can enhance their mobility in soil [54,55]. NPs are absorbed by plants through their seeds, roots, and leaves, regulating nutrient availability to crops via their gradual, controlled release attributes or targeted delivery mechanisms over an extended period to reduce nutrient loss and ensure environmental safety [29]. Additionally, seed pre-treatment with NPs (seed priming) reduces the chances of target NPs dispersion; however, precise skills are needed to avoid toxicity and subsequent germination inhibition [56,57]. Applying zinc oxide (ZnO) NPs as seed priming agents increased seed zinc content and reduced cobalt-induced toxicity by improving antioxidants and promoting plant growth [58]. Zn is the precursor of many enzymes and has a role in plant signaling, specifically under stress conditions. Recently, Mehmood et al. [59] used conventional fertilizer, urea, and NPs (ZnMgO_2_ NPs) in rice. Their results showed that NPs continued to improve growth over a 40–50-day span compared to conventional urea’s 10-day span. They reported a significant enhancement in growth, as indicated by increased seed germination, root and shoot length, α-amylase activity, total phenolic and flavonoid content, and overall antioxidant activity across various concentrations.

#### 4.1.2. Soil Microbial Modulation and Soil Fertility

NPs influence soil microbial communities, fostering beneficial interactions and modulating root morphology to improve nutrient uptake. Saurabh et al. [60] used Fe_2_O_3_ NPs along with *Azotobacter* and farmyard manure on the growth and yield of cauliflower and reported that NPs significantly augment photosynthetic activity and increase enzymatic catalysis in plants. Recently, Upadhyay et al. [61] used conventional fertilizer (NPK) and NPs based fertilizer (nUrea, nZn, nNitrogen) in maize wheat cropping systems. They reported that NPs and N as basal are sustainable nutrient management techniques regarding growth, yield, and rhizosphere biological activity. The foliar spray of nitrogen and/or nZn enhanced the soil microbial community and positively correlated with wheat and maize yield. Similarly, Chen et al. [62] studied the effects of nanocarbon and nanocalcium carbonate on soil enzyme activity and microbial communities. The specific results showed that the individual and combined NPs enhanced soil microbial community and wheat rhizosphere enzymes, with a more profound effect under combined treatments. The relative abundance of microbial communities was positively correlated with soil enzymes. Hence, NPs are crucial in preserving soil fertility and enhancing crop yield, contributing to sustainable agriculture practices [63]. This mechanistic insight is a foundation for developing customized formulations, enabling precise applications in various crops and soil conditions, thus propelling the evolution of precision agriculture [64,65].

**Table 1:** Use of NPs as plant growth stimulators to enhance plant growth and development.

Type	NMs	Concentration	Method	Plant	Duration	Effect on Plant	Ref.
**Metallic-based NPs**	ZnO NPs	50, 100, 150, 200 ppm	Seed priming	*Pennisetum glaucum*	6 h	Seed germination and agronomic traits improved	[66]
	Fe_3_ O_4_ NPs	10, 50, 100 mg L^−1^	Sand culture	*Lycopersicon esculentum*	7, 14 days	Germination, growth, chlorophyll content and SOD improved MDA reduced	[67]
	Fe_2_O_3_ NPs	50, 75, 100% (13 mg plant^−1^)	Foliar and drench	*Raphanus sativus*	weekly	Biomass, chlorophyll, and antioxidants increased, Fe and K content improved in root and shoot	[68]
	ZnO NPs	25, 50, 100 mg L^−1^	Foliar spray, soil irrigation	*Ginkgo biloba*	Every 4 days	25 mg L^−1^ improved biomass, zinc content, and flavonoids, while 50 and 100 mg L^−1^ had an inhibitory effect on plant growth	[69]
	CuO-NPs	25, 50, 75, 100 mg kg^−1^ 20, 40, 60, 80 ppm	Sand Foliar	*Vigna unguiculata*	Foliar 21 days/3rd day	Both soil and foliar application of NPs improved plant morphological attributes, antioxidants, and chlorophyll content	[70]
	Fe_2_O_3_ NPs	100 mg L^−1^ together with inoculant	Soil	*Lactuca sativa*	NM	NPs, together with bioinoculants, increased antioxidant activity, including TEAC, CUPRAC, and DPPH activities in leaves	[71]
	Zn-Nano Ca-nano	1.5 g L^−1^ 2 g L^−1^	Foliar spray	*Arachis hypogea*	30 days	Plant agronomic, yield, and quality-related traits improved	[72]
**Mineral-based NPs**	Molybdenum NPs	6.25, 0.6 mg plant^−1^	Media	*Triticum aestivum*	7 days	Dose-dependent response, high dose caused depressed physiology with robust protein upregulation	[73]
	CaCO_3_ NPs	50, 150, 250 mg L^−1^	Foliar spray	*Lycopersicon esculentum*	10-day intervals	Differential response to concentration, 250 mg L^−1^ increased biomass while 150 and 50 mg L^−1^ improved flower and fruit yield	[74]
	HAP NPs	1000 mg L^−1^	Soil	*Triticum aestivum*	NM	Plant growth and physiological attributes improved in acidic soil	[75]
**Composite NPs**	ZnFe_2_O_4_	5 µM	Soil	*Pisum sativum*	40 days	NPs, together with AMF, improved plant enzymatic activities, metabolites, and nutrient content	[76]
	CuFe-LDHs NPs	1, 10, 100 μg/mL	Foliar spray	*Lactuca sativa*	Twice a week	Plant physiological, photosynthetic, and intercellular CO_2_ increased and modified gene expression patterns	[77]
	Nitrogenous nanocomposite	25, 50, 75% together with CF	Soil	*Lactuca sativa*	45 days	50 and 75% showed improved growth, leaf N, and NO_3_ content as compared to CF	[78]
	Polyherbal nanoformulation	1, 5, 10 ppm	Foliar spray	*Vigna radiata, Trigonella foenum*	NM	The seed germination, plant growth, and chlorophyll contents increased with increasing concentration	[79]

HAP NPs: Hydroxyapatite NPs, NM: Not mentioned.

However, despite the substantial potential of nanotechnology in agriculture, several limitations must be overcome. Optimum concentration and required skills must be acquired before operating to reduce the chances of toxicity. For instance, Gil-Díaz et al. [80] evaluated the foliar effects of two commercial nanofertilizers on spinach plants: Nualgi Foliar Spray, a nanoscale fertilizer combining 12 essential nutrients loaded onto nano-silica, and NovaLand-Nano, a formulation containing nano-sized macro- and microelements designed to promote plant growth. They stated that the NPs had no adverse effect on plant biochemical and ultrastructural attributes at recommended doses, while at higher concentrations, they modified the surface composition, affected the nutrient content of the leaves, and caused ultrastructural damage. Assessing possible ecological effects requires a detailed understanding of how NPs interact with organisms, including plants and soil microbial organisms.

### 4.2. Role of Nanoparticles in Abiotic Stress Tolerance

**Figure 4:**
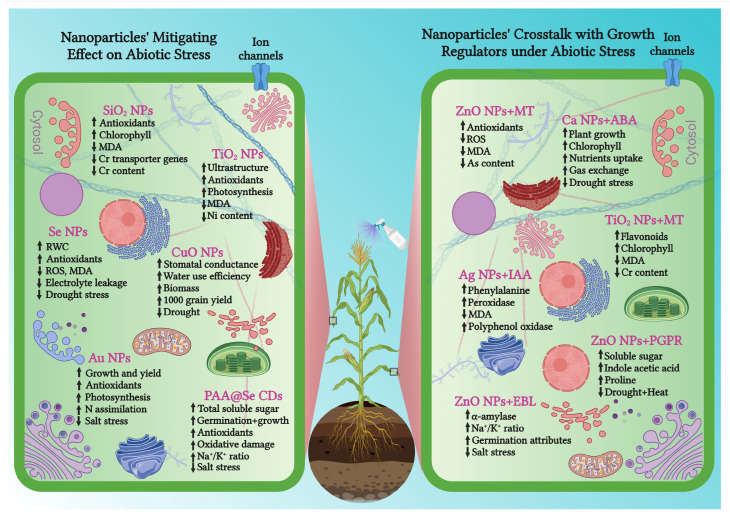
Direct effects of NPs as stress mitigators against various stresses and their synergistic interaction with growth modulators to enhance tolerance.

Abiotic stress, such as HM, drought, cold, and salinity stress, has detrimental effects on crop growth and physiology, leading to reduced yield. Studies have shown that NPs play a dual role by improving plant growth and inducing stress tolerance (Figure 4, Table 2).

#### 4.2.1. Role of Nanoparticles in Inducing Heavy Metal Stress Tolerance

Heavy metal contamination is an increasing environmental and agricultural concern due to the persistence, toxicity, and bioaccumulative nature of metals such as cadmium (Cd), lead (Pb), mercury (Hg), and arsenic (As). These contaminants are mainly introduced through industrial effluents, mining activities, and the excessive use of agrochemicals, leading to severe degradation of soil health and reductions in crop yield [94]. Nanoparticles have been reported to induce HM stress tolerance via various mechanisms.

##### Reduction in Heavy Metal Uptake and Translocation

Huang et al. [95] reported that Si NPs via foliar spray induced Cr tolerance. Their results showed that foliar application reduced Cr uptake by 92% and 76% in roots and leaves, respectively, and increased leaf Si content by 169%. Moreover, Si NPs improved plant growth attributes under stress conditions, such as light-harvesting pigments and gas exchange attributes, and reduced the transcript level of Cr transporter genes. Rehman et al. [96] recently used TiO_2_ NPs via seed priming against nickel (Ni) stress. The results showed that Ni stress caused ultrastructural damage and induced oxidative stress by overproducing ROS and malondialdehyde (MDA). These changes resulted in reduced plant growth and development. In contrast, TiO_2_ NPs priming reduced Ni uptake and increased plant growth and photosynthesis traits. Moreover, the NPs increased antioxidant gene expression and improved plant nutrient uptake under stress conditions. In their study, TiO_2_ NPs are localized in the inner and outer layers of the seed, revealing seed augmentation with TiO_2_ NPs.

##### ROS Scavenging and Antioxidant Defense Activation

Heavy metal stress induces ultrastructural damage by producing ROS and MDA contents, whereas Si NPs activate plant antioxidant enzymes (SOD, POD, CAT, APX, Gr, GSSH) and reduce ROS contents, resulting in plant stress tolerance [95]. In a study, Prakash et al. [97] exogenously applied ZnO NPs under Cr stress and reported that NPs reduced the inhibitory effect of Cr stress and increased rice growth. The specific results showed that Cr stress accumulated ROS and MDA in root tissues and caused oxidative stress. NPs increased plant photosynthetic efficiency and the transcript level of antioxidant genes. The increased antioxidants reduce Cr-induced oxidative stress and augment Cr tolerance. Similarly, foliar application of ZnO NPs reduced Cr-induced toxicity by reducing its uptake and lowering the oxidative stress markers. Specifically, ZnO NPs augmented enzymatic and non-enzymatic antioxidants and increased soybean nutrient profiling [98]. Studies have reported that seed augmentation with NPs brings positive changes in seeds that help plants withstand stress later in the growth stages [57]. Ahmed et al. [99] synthesized Si NPs from rice straw using the green route and used them with bulk Si against Cd stress in *Brassica napus*. The results showed that Cd-stressed plants had the highest in planta Cd concentration with damaged cellular ultrastructure, reduced chlorophyll, and antioxidants. In contrast, Si NPs improved plant photosynthesis and gas exchange attributes, improving plant growth. Specifically, Si NPs reduced Cd uptake and associated damages while increasing plant photosynthesis, antioxidants, and root shoot Si contents. Interestingly, the effect of Si NPs was higher in all studied attributes than in the bulk Si. The findings highlight the superior source-to-sink efficiency of nanomaterials compared to conventional materials. However, there is limited knowledge of NPs-induced tolerance against HM stress at the molecular level. Future research needs to identify downstream stress responses of NPs at the molecular and hormonal levels in different crop species under diverse environmental conditions.

#### 4.2.2. Role of Nanoparticles in Inducing Drought Stress Tolerance

Water scarcity is a major abiotic stress that severely affects agricultural growth and development, accounting for nearly 70% of potential global losses in crop production and productivity. Drought arises from insufficient rainfall or significant imbalances in soil moisture and is widely recognized as one of the most severe abiotic stresses limiting agricultural and forestry productivity worldwide [100].

##### NPs Induced ROS Scavenging and Antioxidant Defense Improvement

Recent studies show NPs reduce drought stress by lowering ROS-related oxidative damage, especially H_2_O_2_ and O_2_^−^, and increase osmolytes and osmoprotectants like prolines, glycine betaine, soluble sugars, and amino acids, aiding osmotic adjustment during drought [101,102]. The beneficial impacts of carbon and metal-based NPs are influenced by their concentration, morphology, surface characteristics, application method, and the specific plant species involved. Se NPs have been reported to increase antioxidants (SOD, CAT, GPX, POD, APX) while reducing electrolyte leakage, ROS, and MDA contents [103]. Se NPs induced increased chlorophyll contents and accessory pigments, which could be attributed to enzymatic activity and chloroplast stabilization, resulting in a maintained cellular integrity. Studies have shown that cerium oxide (CeO_2_) is a potent ROS scavenger and plays a key role in drought and salinity stress. A report by Boora et al. [104] revealed that Ce NPs at 50 PPM concentration rescued wheat from drought stress by reducing ROS (50.53%) and MDA (22.79%). The results showed that NPs induced stress tolerance by increasing proline content, RWC, and photosynthesis. The NPs upregulated stress-responsive genes (*MYB33*, *MYB3R*, *DREB2*, *ABC1*, and *SnRK2.4*), increasing antioxidant activities. Another study reported that Ce NPs augmented stress tolerance in grapevine by improving water potential and agronomic attributes [105]. Ce NPs at 50 mg L^−1^ reduced the oxidative stress by highly increasing the transcript level of antioxidant genes (*VvCLH1*, *VvCu/ZnSOD*, *VvRD29A*, *VvRBOHA*), which reduced H_2_O_2_ (32.63%), EL (40.35%) and MDA (50.63%). However, the underlying molecular mechanism that brought these changes is yet to be elucidated, and additional research is needed for novel biogenic NPs in valuable crops under water stress conditions.

##### NPs Induced Osmotic Adjustment and Water Status Regulation

In a study, Zeeshan et al. [103] applied Se NPs via a foliar way and found improved relative water contents (RWC) and photosynthesis attributes under stress. CuO NPs at 300 mg L^−1^ improved wheat growth regarding stomatal conductance, leaf chlorophyll contents, plant biomass, and 1000-grain yield [106]. Interestingly, NPs also improved leaf turgor pressure, water use efficiency, and RWC. However, increasing concentration (700, 950 mg L^−1^) caused adverse effects and reduced growth attributes. Recently, Javan et al. [107] evaluated the drought-mitigating efficiency of TiO_2_ NPs in strawberries. The study found that a 30 mg L^−1^ of NPs spray reduced sustained deficit irrigation by 25.2% and increased the strawberry agronomic attributes (leaf number and area, fruit length, weight, diameter) and biochemical attributes (carbohydrates, phenol, flavonoids, etc.).

#### 4.2.3. Role of Nanoparticles in Inducing Salt Stress Tolerance

Soils containing excessive soluble salts or high levels of exchangeable sodium (Na^+^) in the root zone are classified as soil salinity [108]. It poses a persistent threat to crop health and productivity by disrupting physiological processes, altering nutrient balance, and reducing growth and yield [109]. Soil salinity has impacted approximately 830 million hectares of arable land, significantly constraining crop development and productivity [110]. Interestingly, studies have shown that an optimum use of NPs can reduce their adverse effect on plant growth and metabolism and consequently improve yield [111].

##### Osmotic Adjustment and Ion Homeostasis Regulation

Salinity stress induces osmotic imbalance and ion toxicity, particularly due to excessive sodium accumulation. Under osmotic stress, proline accumulation in plants is a defensive response to overcome the stress effect. It is a key osmolyte and plays a crucial role in plant osmotic homeostasis. Fouda et al. [112] studied that CuO NPs increase plant proline contents and reduce ROS and MDA contents under salt stress. They stated that as a single regulatory molecule, proline activates several reactions associated with plant adaptation. NPs regulate the K^+^/Na^+^ ratio, decreasing sodium ions while increasing other essential ions [113], activating plant antioxidants, and restoring photosynthesis [114]. Similarly, gold (Au) NPs have been reported to reduce salt-induced abnormalities in crops by upregulating antioxidant defense mechanisms and balancing sugar and nitrogen metabolism. Song et al. [115] reported that astaxanthin-synthesized Au NPs increase tetrapyrrole biosynthesis and the transcript level of ROS scavenging genes and increase antioxidants under salt stress. Astaxanthin is a naturally occurring antioxidative oxycarotenoid present in many shellfish and microorganisms and works as a potent antioxidant agent against oxidative stress.

##### Enhancement of Growth, Photosynthesis and Metabolism

Nanoparticles also alleviate salinity stress by improving photosynthetic efficiency, metabolic processes, and early developmental stages. Biogenic ZnO NPs enhanced chlorophyll content and phenolic compounds in capsicum, leading to improved photosynthetic performance under salt stress [116]. ZnO NPs boost crop resistance to environmental shocks and promote global efforts to apply eco-friendly agricultural technologies that maintain natural resources and ecological balance. Gao et al. [117] used lanthanum oxide (La_2_O_3_) NPs as a seed-priming agent in alfalfa and reported significantly enhanced seed germination under saline conditions. The NPs ruptured the seed coat and increased α-amylase activity, facilitating rapid water uptake and reducing salt-induced inhibition during germination. Khatoon et al. [118] used biogenic Au NPs synthesized from wheat leaves in mustard under salt stress. Their results showed that the NPs reduced salt stress by increasing antioxidants and reducing MDA and ROS. Consequently, the chlorophyll, sugar, and nitrogen metabolism improved, resulting in an increased 1000-grain yield.

#### 4.2.4. Role of Nanoparticles in Inducing Combined Stress Tolerance

Combined stress, arising from the simultaneous occurrence of multiple abiotic stresses such as drought, salinity, HMs, and extreme temperatures, disrupts plant growth, reduces productivity, and threatens agricultural sustainability [119]. For instance, drought and salinity mainly occur together and are the two most significant environmental conditions that reduce agricultural output globally. Interestingly, recent studies reported that NPs play a crucial role in mitigating the combined effect of these stressors.

##### ROS Scavenging and Antioxidant Defense

Khan et al. [120] reported that Si NPs mitigated the combined effects of drought and salinity by enhancing antioxidant activities and osmolyte accumulation while reducing electrolyte leakage and MDA content. These changes contributed to improved cellular stability and stress tolerance. Similarly, Al–Mayahi [121] demonstrated that Fe_2_O_3_ NPs, in combination with salicylic acid (SA), significantly reduced oxidative damage under dual drought–salinity stress by increasing antioxidant enzyme activity and chlorophyll content while lowering Na^+^ and MDA levels. Under combined Cd and drought stress, Adrees et al. [122] reported that iron NPs via soil application reduced ROS accumulation, electrolyte leakage, and MDA content in wheat while enhancing antioxidant capacity and chlorophyll levels. Comparable findings were reported by Ahmed et al. [123], where Fe NPs combined with hydrogel NPs improved antioxidant activity and reduced ROS accumulation under Cd–drought stress in rice.

##### Osmotic Regulation, Ion Homeostasis, and Nutrient Balance

Nanoparticles play a crucial role in maintaining osmotic balance and regulating ion homeostasis under combined stress conditions. Khan et al. [120] demonstrated that Si NPs restored K^+^/Na^+^ homeostasis under drought and salinity stress by reducing Na^+^ accumulation while improving osmolyte levels, sugar content, and chlorophyll concentration. Biogenic Si NPs were also reported to alleviate salinity stress in HM-contaminated soils in a two-year field experiment [124]. The results showed enhanced uptake of essential nutrients (N, P, K, Ca^2+^) and improved K^+^/Na^+^ ratios, leading to increased plant growth. Notably, these NPs significantly reduced the uptake of Na^+^, Pb, Ni, and Cd in leaves and pods, thereby lowering electrolyte leakage, ROS, and MDA levels. Under Cd–drought stress, Ahmed et al. [123] observed that Fe NPs and hydrogel NPs reduced acropetal translocation of Cd and downregulated Cd transporter genes (*OsHMA2*, *OsHMA3*, *OsLCT1*), highlighting a molecular basis for improved ion regulation and reduced metal toxicity.

##### Regulation of Photosynthesis, Growth, and Stress-Responsive Gene Expression

Nanoparticles also enhance combined stress tolerance by improving photosynthetic performance, growth traits, and gene expression associated with stress adaptation. Toaiema and Mustafa [125] reported that Si NPs enhanced vegetative growth parameters, including root and shoot length, biomass, and volatile oil content under combined stress conditions. However, the authors emphasized the need for molecular-level investigations to link phenotypic responses with underlying regulatory mechanisms. Selenium is a potent metalloid with metal-like properties, and its NPs have been largely reported to augment drought and heat stress tolerance. In a study involving six wheat varieties, Se NPs significantly enhanced growth attributes, antioxidant activity (SOD, POD, CAT), and gas exchange parameters, while reducing H_2_O_2_ and MDA levels under dual stress conditions [126]. Importantly, Se NPs upregulated stress-responsive genes under both drought and heat stress. In another study, El-Saadony et al. [127] synthesized biogenic Se NPs using *Lactobacillus acidophilus* and reported their dual role in mitigating biotic and abiotic stress. These NPs significantly reduced wheat crown and root rot caused by *Fusarium* spp. while improving plant growth and grain yield. Although drought and heat stress tolerance were claimed, supporting physiological or molecular data for these conditions were not provided. Additionally, Dinler et al. [128] demonstrated that seed priming with TiO_2_ NPs improved sunflower growth and yield under combined drought and salinity stress by stimulating antioxidant defense mechanisms and preserving membrane integrity. However, further studies are required to validate these findings across later developmental stages and yield-related traits.

### 4.3. Nanoparticles’ Interaction with Nanoparticles/Growth Regulators to Improve Stress Tolerance

Studies have shown that the interaction (synergistic or antagonistic) between NPs and phytohormones significantly influences plant growth and defensive responses under various environmental conditions (Figure 4). It is reported that NPs influence the levels of various phytohormones, thereby impacting plant growth and defense mechanisms [89,129]. A recent study elaborated on the combined efficacy of ZnO NPs and melatonin (MT) for As tolerance in soybean plants [130]. Both ZnO NPs and MT, when applied individually or in combination, enhanced As tolerance in soybean seedlings. However, their combined application proved more effective than either treatment alone. The findings revealed that ZnO NPs and MT synergistically activated the antioxidant defense system and reduced the accumulation of MDA and H_2_O_2_ in the seedlings. Likewise, the co-application of silver (Ag) NPs and indole acetic acid (IAA) enhanced plant growth by lessening the concentration of MDA [131]. Ag NPs and IAA increased the activity of phenol-synthesizing and oxidizing enzymes such as peroxidase, phenylalanine ammonia-lyase, and polyphenol oxidase. Furthermore, research indicates that adding TiO_2_ NPs and MT improved the drought resilience of Stevia (*Stevia rebaudiana* Bertoni) plants under moderate to severe stress conditions. They enhanced the growth traits and photosynthetic activities in seedlings under drought conditions [132]. The TiO_2_ NPs and MT combination may mitigate Cr toxicity by enhancing chlorophyll level, total flavonoid levels, and total phenolic content in lemon balm [133]. However, further studies on NPs and MT application and dosage methods are required to thoroughly assess their potential in environmental and agricultural applications. Guardiola-Márquez et al. [134] used Fe and Zn as NPs with biofertilizers and reported a significant increase in agronomic attributes. Their finding revealed that Fe and Zn NPs synergized with biofertilizers and identified 8 *glucosinolates* to improve their interaction significantly.

The combination of TiO_2_ NPs at a concentration of 2000 mg kg^−1^ and the nitric oxide donor sodium nitroprusside (SNP) at 100 μM was observed to improve seedling length, SOD, total soluble proteins, net photosynthetic rate, transpiration, and intercellular CO_2_ concentration [135]. TiO_2_ NPs function as NPs and promote biomass accumulation by activating several plant metabolic processes [136]. Nitric oxide is crucial in regulating phytohormones, such as cytokinin, auxin, and gibberellin, essential for tissue differentiation, cell elongation, and cell division, facilitating optimal plant growth under stressful situations. It has been reported that the synergistic interaction of TiO_2_ NPs and calcium phosphate enhanced the membrane stability index (MSI), RWC, chlorophyll content, osmolyte levels (sugar and proline), and the antioxidant defense system (SOD, POD, CAT). Soliman et al. [137] stated that selenium-chitosan (Se-NPs) and biochar (SB) can protect wheat plants from salt-induced damage. Plants subjected to NaCl +  SB  +  Se-NPs exhibited a 50% reduction in Na^+^ content in leaves relative to those treated with NaCl alone. They concluded that Se NPs and SB can restore carbon assimilation and ion homeostasis in salt-stressed wheat by increasing the expression level of key transporter genes in the leaves. Se-NP and SB regulated many antioxidant enzymes to protect wheat growth and physiological functions under salinity stress. Se-NP and SB markedly enhanced the levels of flavonoids and phenols in salt-stressed wheat, accompanied by a rise in osmolytes such as betaine, glycine, proline, and carbohydrates. The improved levels of flavonoids and phenols boosted the antioxidant system, consequently mitigating the negative impact of ROS on cellular organelles. Zahedi et al. [129] indicated an enhanced production of secondary metabolites in plants by applying Se-NPs. Similarly, Ciccolini et al. [138] found that increased flavonoids and phenols generated by SB in lettuce enhanced antioxidant efficacy and stress mitigation. The combined application of co-composted biochar and ZnO NPs was investigated under Cd and water-limited stress in wheat plants [139]. The results showed increased biomass and chlorophyll pigments by modifying the antioxidant enzyme activities and reducing plant oxidative stress. Additionally, the co-application inhibited the Cd uptake in grains below the threshold level, which may have contributed to the increase in biomass in wheat plants. The combined biochar and ZnO NPs supply improves plant growth under Cd and drought stress. However, molecular analysis is still necessary to comprehensively understand the detailed mechanisms.

A study investigated the impact of ZnO NPs synthesized using an extract of Papaya fruit and used with PGPR under heat and drought stress [140]. Their results demonstrate that the defense mechanisms of plants are improved against drought and heat and their combined stress after the co-application of ZnO NPs and PGPR (*Pseudomonas* sp.). The NPs and PGPR alleviated stress by enhancing the nutrient contents, IAA, soluble sugar, and protein and improving the photosynthetic pigments and plant biomass. The simultaneous exposure of NPs and PGPR enhanced proline levels and augmented antioxidant defense mechanisms, including SOD, POD, APX, CAT, dehydroascorbate reductase (DHAR), glutathione reductase, and abscisic acid (ABA) and reduced electrolyte leakage, H_2_O_2_, and MDA levels. These approaches may offer sustainable mitigation platforms for HMs in soil–plant systems. A study examined the beneficial effects of *Bacillus fortis* IAGS 223 and ZnO NPs on *Cucumis melo* plants under Cd exposure [141]. Their findings indicated that the concurrent application of ZnO NPs and PGPB significantly reduced oxidative stress markers, including MDA and H_2_O_2_, in plants subjected to Cd stress. Moreover, utilizing ZnO NPs and PGPB led to a reduction in Cd concentrations in plant shoots. Recently, Liu et al. [142] co-applied Se and CuO NPs to strawberries under drought-stress conditions. NPs improved the agronomic trait indices and water use efficiency while reducing drought-induced adverse effects. Additionally, NPs increased photosynthetic traits (chlorophyll a, b, etc.) and antioxidant defense (SOD, POD, CAT) and reduced MDA content. Se NPs alone demonstrated greater efficacy than CuO NPs alone, and combining both yielded the highest effectiveness. Table 3 lists the recent studies reporting synergistic interaction of NPs with plant hormones in alleviating abiotic stress. However, these findings need to be validated under natural field conditions. Additionally, molecular studies are crucial to fully understanding the coordinated mechanisms of NPs and growth regulators in mitigating environmental stress in edible crops.

### 4.4. Strategies to Enhance the Beneficial Effects of Nanoparticles in Plants

The beneficial effects of NPs on plant growth and stress tolerance depend on several controllable factors, including surface properties, composition, NPs size, concentration, and application method. Therefore, modifying NPs attributes can be an effective strategy to enhance their beneficial efficiency in crop plants. Surface coatings are critical in regulating NPs’ toxicity, stability, solubility, and biocompatibility. NPs typically comprise a core of varying size and shape, modified with surface coatings to enable functionalization for diverse applications [152]. Surface modification improves biodistribution, enhances cellular uptake, and influences subcellular localization, largely through effects on surface charge (zeta potential) [153]. Positively charged NPs generally show higher cellular uptake than negatively charged ones. Uncoated NPs often exhibit significant toxicity at high concentration, whereas appropriate coatings reduce adverse biological effects and improve stability by preventing aggregation. Although citrate coatings are commonly used for metallic NPs, particularly gold, their high zeta potential can promote aggregation; therefore, alternative coatings, such as silica and other biocompatible materials, are widely employed to enhance stability and compatibility [154]. These nanoscale materials possess a high surface-area-to-volume ratio, which enhances their reactivity and interactions with plant systems. This property enables them to penetrate plant cell walls and efficiently adsorb nutrients and agrochemicals, thereby improving their transport and bioavailability in plants. The size of NPs plays a critical role in determining their uptake, internal transport within plants, and interactions with plant cellular components. NPs size can be precisely tuned through a combination of chemical factors (such as pH, surfactants, and stabilizers), physical approaches (e.g., laser ablation and sputtering), biological routes, mechanical processing, and post-synthesis modifications. Integrating these strategies enables the production of NPs with tailored sizes, thereby optimizing their performance in practical applications [155]. Kim et al. reported that ZnO NPs sized 10–30 nm accumulated more efficiently in the leaves of *Brassica chinensis* L. (bok choy) than larger 300 nm particles, which, while less mobile, exhibited greater toxicity [156]. NPs in the 3–5 nm size range are particularly effective at overcoming physical barriers such as the cuticle and root epidermis and demonstrate increased mobility across the plant’s vascular system [42]. The bioavailability of nanomaterials depends largely on their solubility in aqueous and soil environments [157]. In contrast, engineered NPs with controlled solubility can provide a sustained release of nutrients, reducing application frequency and minimizing environmental impacts [158]. Kumari et al. [159] stated that high NPs concentrations (up to 2000 mg L^−1^) can adversely affect plant biochemistry, morphology, and physiology and may even induce genotoxic effects, whereas application at properly optimized and standardized doses generally results in beneficial outcomes. NPs may act synergistically with rhizosphere microbes to enhance nutrient cycling, stimulate microbial activity, improve plant nutrient uptake efficiency, and ultimately strengthen plant growth and stress tolerance [160]. The application method of NPs greatly influences their efficiency, although the optimal approach depends on the desired plant response. For instance, Zhao et al. applied 50 nm ZnO NPs at 50 mg/L and reported that seed priming most effectively enhanced seed germination (51.22% increase) and seedling growth (64.27% increase in seedling height), whereas foliar application of the same NPs significantly promoted the growth of *H. syriacus* seedlings, with increases of 248.83%, 281.92%, and 97.90% in root, stem, and leaf biomass, respectively [161]. Overall, by carefully optimizing NPs properties, concentration, and application methods, it is possible to maximize their beneficial effects on plant growth, nutrient uptake, and stress tolerance, while minimizing toxicity and environmental risks.

## 5. Nanoparticles in Pollutant Remediation: Reducing Source-Sink Dynamics

**Figure 5:**
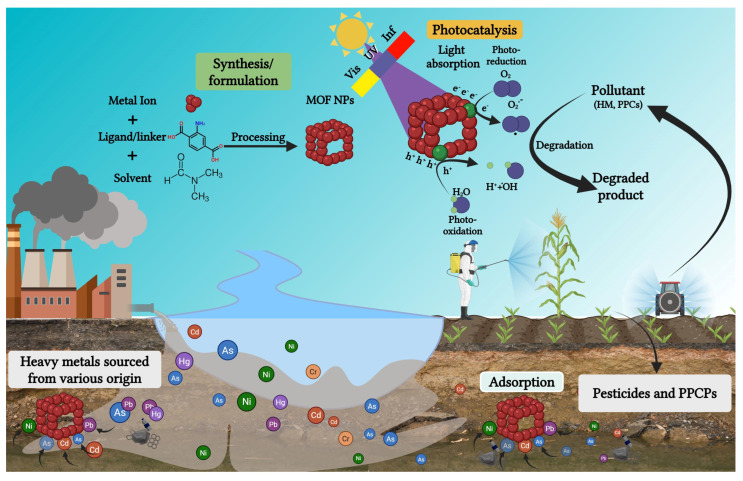
Nanoparticles exhibit diverse techniques, including photocatalysis and adsorption, to effectively cleanse water and soil of contaminants such as HMs (HM), pharmaceuticals and personal care products (PPCPs), and pesticides.

Nanoparticles have emerged as promising agents in addressing environmental challenges, particularly pollutant remediation. By targeting source-sink dynamics, they play a pivotal role in reducing the accumulation and mobility of contaminants in soil and water systems. Various studies have found different strategies for remediating soil, groundwater, wastewater, and landfill leachate contaminated by other sources [162,163,164]. This approach minimizes pollutant availability for plant uptake, safeguarding crop health and environmental quality [165]. For instance, Yang et al. [166] developed biochar-loaded cobalt NPs with uniform dispersion and ultra-high stability. In their study, the cobalt NPCs (Co-CGBC-700) showed excellent efficiency and durability in catalyzing Fenton-like reactions to degrade various organic contaminants. The catalytic activity of the NPs in the practical treatment of polluted water and soil showed high degradation efficiency (>90% in 60 min) for environmental applications. Figure 5 shows a schematic illustration of how NPs can be used as remediating agents for the pollutants. Another study by Xie et al. [167] developed zero-valent iron modified with carboxymethyl cellulose and biochar (CMC-nZVI/BC) to treat Cr-contaminated soil. Their findings showed that in 90 days, the NPs and biochar effectively immobilized 96.6% and 97.0% in high and low-contaminated soil, respectively. They further stated that CMC-nZVI/BC could effectively immobilize around 85% in 400 h at a concentration of 40 mg/L at a flow rate of 0.5 mL/min. Similarly, Vojoudi et al. [168] reported a new nano-adsorbent synthesized by encapsulating a magnetic Fe_3_O_4_ core with a mesoporous silica shell as an adsorbing agent. They stated that 303, 256.4, and 270.3 mg/g of Hg(II), Pd(II) and Pb(II) ions were removed from water, respectively. Fe oxide NPs synthesized from tea leaf extracts have been reported to remove 13.7 mg/g As(V) from contaminated water [169]. Sithara et al. [170] synthesized biogenic Fe_2_O_3_ NPs from orange peels and reported their dye degradation potential in a contaminated environment. Their specific results revealed that the NPs demonstrated a photocatalytic remediation efficiency of 97% for methylene blue under visible light irradiation, exhibiting concentration-dependent biofilm inhibition against *E. coli* and *S. aureus*. The study found that Fe_2_O_3_ NPs effectively purify water, inhibit pathogens, and are reusable due to their degradation activity and biofilm inhibition. Another study synthesized a multifunctional SrTiO_3_/Ag nanocomposite that serves as both a supercapacitor material and an effective photocatalyst. According to their findings, the nanocomposite had an outstanding methylene blue dye degradation efficiency of over 88% following 120 min of UV irradiation [171]. Similar studies reported remediation and immobilization of toxic substances in soils using GO, CNTs, and nZVI [172,173,174]. Arenas-Lago et al. [175] reported that calcium phosphate NPs (CPNs) effectively immobilized HMs, including Pb, Cu, and Zn, in shooting range soils. The application of these NPs in the soil resulted in a reduction in Pb and Cu levels by over 90% and Zn levels by 50%. Likewise, nano-hydroxyapatite decreased Pb(II) levels in Ryegrass by 2.86–21.1% in the roots and 13.19–20.3% in the shoots [176]. This reduction might be attributed to the secretion of tartaric acid in the root rhizosphere, which promoted the adsorption of Pb onto the NPs. Iron-based MOF has the most potential to remediate almost all water contamination because of its low toxicity, high porous structure, and stability [177]. Table 4 lists recently employed NPs used for water and soil remediation, detailing their mechanisms of action. Nanotechnology supports environmental sustainability by promoting the use of renewable energy sources and enabling the recovery and recycling of resources from wastewater. However, to fully realize its potential in wastewater remediation and public health improvement, responsible development, robust regulation, and increased public awareness are essential.

## 6. Environmental Risks Associated with the Use of NPs and Their Management

**Figure 6:**
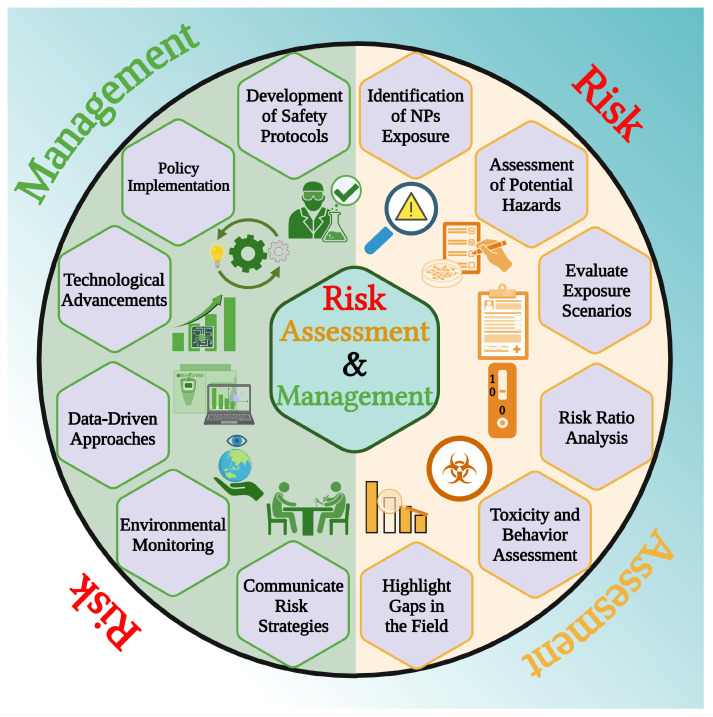
This schematic diagram shows the key steps involved in assessing the risk associated with the use of NPs and their subsequent management, ensuring safe and effective applications in agrochemicals while addressing environmental and health concerns.

In previous discussions, we highlighted that those manipulating materials at the nanoscale have emerged as a promising strategy with wide-ranging applications in agriculture. Although the application of nanotechnology in agriculture offers significant benefits, such as enhanced solubility, bioavailability, targeted delivery, reduced chemical pollution, and improved crop productivity through nano-fertilizers and nano-pesticides. However, its adoption is limited by ethical and regulatory challenges, particularly concerns over potential risks to human health, ecosystems, and aquatic life. The cellular interactions and long-term effects of engineered NPs are not yet fully understood, highlighting the need for thorough risk assessments and robust regulatory frameworks [193]. Although the concerns and challenges discussed henceforth are primarily anticipatory based on emerging research and projected trends, they highlight the need for comprehensive studies and robust regulatory oversight to ensure the safe and effective application of nanotechnology in agriculture (Figure 6).

### 6.1. Risk Assessment

The assessment begins by identifying the NPs and understanding their potential exposure conditions. A comprehensive risk assessment is necessary to understand the potential hazards, likelihood of exposure, and resulting risks to people and plants associated with NPs. However, there is a lack of knowledge in the current literature regarding the risk assessment of NPs in agriculture. Most research has adapted risk assessment methodologies from other compounds, such as pharmaceuticals or synthetic chemicals [194]. There are no established procedures for evaluating agrochemicals based on nanomaterials, and there is still no established technique despite several assays that have been suggested. Therefore, it is challenging to compare the findings of agrochemicals based on nanomaterials and come to a consensus regarding their toxicity [195]. Currently, no nation’s government has established a set of toxicity regulating plans for NPs that are used to evaluate the safety, health, and ecological effects of these particles for both humans and animals [194]. NPs are minute in size and have unique physical properties, making them cross cellular barriers and accumulate in plant/human tissues. Here, the concentration of NPs plays a crucial role and should be monitored precisely. A higher concentration of NPs interacts with cellular compartments, disrupting cellular processes, generating ROS, and interfering with cell division mechanisms by binding to proteins and inhibiting protein synthesis. Secondly, NPs may pose toxicological risks to individuals involved in their large-scale synthesis or application. For example, certain NPs have been shown to disrupt mitochondrial function by altering cell membrane permeability to K^+^ and Na^+^ ions. They can also exert toxic effects on the proliferation and cytokine expression of peripheral blood mononuclear cells (PBMCs), as well as negatively impact the male reproductive system in individuals directly exposed to NP production, application, or bioaccumulation [196]. Moreover, assessments of potential hazards should be grounded in empirical data rather than guesswork/estimation. Identifying various environmental components and potential exposure routes is crucial to assessing prospective exposure situations. The Predicted No-Effect Concentration (PNEC) is determined following the identification of hazards and is based on assessing the risk associated with the PNEC. To identify potential hazards sourced from NPs, a risk ratio is established through several procedures to identify potential dangers. A risk ratio is defined as the ratio between the predicted environmental concentration (PEC) of a substance and the PNEC, which represents the level below which no adverse effects are expected. When the risk ratio exceeds one, it indicates that the substance may pose a potential risk to exposed organisms [197]. Risk assessment of the potential dangers associated with NPs has been carried out for a long time, and there has been a certain degree of alteration in the perception of these risks. For instance, Ag-based NPs, commonly employed for their antimicrobial properties, were previously believed to have significant environmental hazards. However, recent findings indicate that these NPs pose a minimal overall risk to terrestrial ecosystems. This is due to several factors: (1) only a small portion of Ag NPs ultimately enter the soil, (2) the nano-properties and activities of Ag NPs rapidly diminish in the soil, and (3) there is only minor bioaccumulation of Ag in edible plant parts [198]. Scientists have also recommended directly using fertilizers and pesticides based on NPs (such as Fe, Cu, Mg, Mn and Si) on the soil or plants. These NPs have minimal environmental risks and promote crop growth [199,200]. However, different types of NPs may have varying effects, and it is essential to conduct a thorough study of these risks before using them specifically for environmental remediation purposes. Ruffatto et al. [201] reviewed that metal-based NPs can accumulate in soil and water, potentially impacting non-target organisms and microbial communities. For example, Cu-NPs have been shown to accumulate in plant tissues, causing phytotoxicity, oxidative stress, membrane damage, and altered expression of Cu-Zn-SOD genes, as observed in cucumber plants. When applying NPs to plants, several factors must be carefully considered, including particle size, dosage, duration of exposure, surface chemical composition, potential immunological responses, and strategies for managing toxicity. Risk assessment in nanotechnology is particularly challenging because conventional chemical risk assessment assumptions, testing procedures, and modeling frameworks are often inadequate for accurately evaluating the behavior of NPs in the environment and their potential uptake by humans [202].

### 6.2. Risk Management

Following the completion of a risk assessment of NPs, it becomes necessary to implement appropriate risk management techniques. Despite concerns regarding the environmental, health, and safety implications of NPs, governments worldwide acknowledge their substantial potential benefits in various domains. The toxicological assessment of nanomaterials in plant treatment is critical in identifying hazards associated with their use. These include technological advancements, the development of self-cleaning surfaces, enhanced energy efficiency, reduced reliance on specific chemicals, substituting highly toxic substances, and the remediation of contaminated sites. Additionally, it is crucial to adhere to appropriate safety measures when applying nano-agrochemicals. Governments and scientific organizations widely acknowledge the significance of risk management for nanomaterials in agrochemicals [203]. Effective risk communication involves the transparent dissemination of information regarding NPs exposure, potential dangers, appropriate work protocols, protective and preventative measures, and policies to enhance the management of hazards and potential threats. Moreover, implementing the “safety by design” principle is followed to mitigate or eliminate the potential risks associated with the hazards posed by NPs. The US Environmental Defense Fund has recommended researching to assess potential hazards throughout the life cycle of nano products, considering consumer usage, employee safety, and environmental impacts resulting from product disposal. However, the need for field data presents a key obstacle to using nano agrochemicals. The growing knowledge of the uses of nano agrochemicals has yet to encourage the translation of laboratory results into field data. The absence of sufficient data leads to substantial consumer and environmental safety uncertainties, crucial for fostering public trust in products [204]. Environmental fate and exposure measurement should be conducted consistently throughout the experiment, tests, and modeling processes. To reap the benefits of nanotechnology in agriculture, a well-coordinated risk management strategy and a suitably responsive approach are needed. The data from such tests should be evaluated based on the NPs’ exposure and transformation potential during their life cycle. To ensure the safe, ethical, and sustainable use of nanotechnology in agriculture and food systems, further research, responsible innovation, and the establishment of regulatory agencies are essential.

## 7. Conclusions and Prospects

Nanotechnology presents a range of potential remedies for current agricultural challenges, addressing concerns arising from climate change and plant diseases to safeguarding food security. By harnessing the potential of NPs, we are moving towards more environmentally friendly solutions that improve plant resilience and nutrient absorption. NPs are effective carriers for targeted agrochemicals, reducing waste generation and environmental contamination. The proper integration of technology needs collaboration among scientists, politicians, and industry executives. The combination of nanotechnology and precision agriculture holds great potential for making substantial advances in the sustainability and productivity of crops. This all-inclusive strategy involves the execution of research, the cultivation of innovation, and the promotion of collaboration. By actively engaging with stakeholders, we establish a path toward a future where food security is strengthened, the utilization of the environment is maximized, and future generations flourish despite shifting climatic conditions.

Nevertheless, there are emerging concerns about the potential ecological impacts of nanotechnology in agriculture. While current findings are largely based on laboratory studies, field-level applications and commercialization remain limited. As such, the challenges discussed are primarily anticipatory, reflecting early-stage research and projected trends. Ongoing risk assessments and the development of proactive control measures will be essential to guide the safe and effective integration of nanotechnology into agricultural practices. Addressing future challenges, such as establishing global standards, improving public acceptance, and overcoming plant-level limitations, will be essential to fully harness the potential of NPs, given their unique properties and the diversity of plant systems. Additionally, it is important to carefully choose materials, preferring natural materials that have minimal impact on the ecosystem’s overall health. Given that numerous components within NPs consist mostly of HMs, conducting additional research on their potential cellular toxicity and genotoxicity, and establishing their threshold levels is imperative. Also, the changes these NPs induce in plants must be unraveled at the molecular level, and their underlying pathways elucidated.

## 8. Research Gaps and Recommendation

Biologically synthesized NPs face stability and degradability challenges in the environment, which can be mitigated by combining them with other molecules. Thus, understanding plant–NP interactions and optimizing NP properties such as size, concentration, and biocompatibility is essential for safe and practical field application.

In recent years, numerous studies have demonstrated the effectiveness of NPs in enhancing abiotic and biotic stress tolerance in edible crops, though most have been limited to laboratory settings. However, concerns remain regarding their environmental impact and accumulation in edible plant parts. Therefore, targeted research is needed to develop appropriate evaluation methods for assessing the effects of NPs on both biotic and abiotic components of ecosystems.

Although NPs have shown stress-mitigating effects in plants, the underlying molecular, transcriptomic, and proteomic mechanisms remain largely unexplored. Future studies at these levels are essential to uncover the pathways involved in NP-induced stress tolerance in edible crops.

Future research should focus on understanding how NPs affect soil microorganisms and the phyllosphere, their impacts on non-target organisms, and the regulatory roles of root exudates and rhizosphere microbes. Additionally, exploring the synergistic interactions between NPs and MAPK signaling under stress conditions is crucial for improving the effectiveness of nanoformulations in sustainable agriculture.

## Figures and Tables

**Table 2:** Use of NPs as a stress mitigation agent to induce stress tolerance and improve plant growth.

Type	NMs	Stress	Size (nm)	Concentration	Method	Target Plant	Effect on Plant	Ref.
**Nano-Iron**	Coated Fe-NPs	Salt	21–30	5, 10, and 15 μM L^−1^	NM	*Trachyspemum ammi*	Increased growth attributes and nutrients, while reduced Na contents	[81]
	Fe_3_O_4_ NPs	Drought	NM	25, 50, 100, 150 mg L^−1^	Foliar spray	*Brassica napus*	improved mesophyll ultrastructure, PSI and PSII efficiency and antioxidants while reducing ROS and MDA	[82]
**Nano-Zinc**	ZnO NPs	Co	20	500 mg L^−1^	Seed priming	*Zea Mays*	Increased seed zinc contents, improved ultrastructure and photosynthesis, and conferred tolerance against cobalt	[58]
	ZnO NPs	Cd	30–70	50 and 100 mg L^−1^	Foliar spray	*Brassica parachinensis*	NPs improved photosynthesis under stress conditions, and 481 untargeted metabolites were enriched in leaves, attributed to various compounds	[83]
**Nano-Selenium**	Se NPs	Salt	20–40	25, 50 mg L^−1^	Foliar spray	*Citrus Limon*	NPs at 50 mg L^−1^ improved chlorophyll, carotenoids, antioxidants and growth attributes while reducing osmotic stress markers	[84]
	Se NPs	Salt	37	0.01%, 0.05%, and 0.1%	Foliar spray	*Triticum aestivum*	0.1% NPs improved chlorophyll and reduced ROS and MDA, consequently increased grain yield	[85]
	Se NPs	Cd	403, 804	15 mg L^−1^	Soil mixed	*Oryza sativa*	NPs activated stress-responsive and signaling pathways, particularly the GA pathway, by 4.79-fold. NPs increased the beneficial endophyte community and biomass by 100%	[86]
**Nano-Silicon**	SiO_2_ NPs	Drought	40	50, 100, 200, and 500 mg L^−1^	Foliar spray	*Ehretia macrophylla*	NPs at 100 mg L^−1^ reduced MDA and ROS, increased antioxidants, and RNA seq analysis showed upregulation of key stress-responsive pathways	[87]
	Si NPs	Drought	NM	300, 600 or 900 mg L^−1^	Nutrient media	*Oryza sativa*	NPs at 600 mg L^−1^ enhanced histological features in the root, improved antioxidants and protein contents, and reduced ROS and MDA	[88]
**Nano-copper**	Cu NPs	Cd	NM	50, 100 and 200 mg L^−1^	Foliar spray	*Solanum melongena*	Low concentrations of NPs reduced oxidative stress by reducing ROS and MDA while increasing soluble protein and RWC	[89]
**Nanotitanium**	TiO_2_ NPs	Cr(IV)	08–30	NM	Foliar spray	*Helianthus annuus*	NPs reduced oxidative stress markers and improved photosynthesis and antioxidant defense systems	[90]
**Nano-Silver**	Ag NPs	Salt	NM	15, 25, 35, 45, and 55 ppm	Foliar	*Pennisetum glaucum*	NPs improved chlorophyll pigments, osmolytes, and antioxidant mechanisms while reducing ROS	[91]
**Nano-Sulfur**	SNPs	Hg	47	300 mg kg^−1^	Soil mixed	*Brassica napus*	NPs restored the relative abundance of soil microflora, reversed Hg-induced changes and restored soil health, improved plant growth	[92]
**Nano-Manganese**	MnO NPs	Pb	22	50 ppm	Seed priming	*Triticum aestivum*	NPs improved leaf and shoot size, root length, chlorophyll and carotenoids, and RWC while decreasing electrolyte leakage and MDA	[93]

NM: not mentioned.

**Table 3:** Nanoparticles interact synergistically with phytohormones to reduce abiotic stress-induced toxicity in various plants.

Type	Synergism	Stress	Concentration	Method	Plant	Response	Ref.
**Metal + hormone**	ZnO NPs + SA	Salt	20 mg L^−1^ + 500 µM	Foliar spray	*Salvia virgata*	The combination treatment enhanced proline, sugar content, and antioxidant levels while lowering MDA and H_2_O_2_, thereby improving the plant’s salt stress tolerance	[143]
	Ag NPs + MT	Drought	0.06 mg L^−1^	Seed priming	*Eugenia uniflora*	Combined application improved germination, seed vigor index and antioxidant by 100%, proline, protein and proline dehydrogenase by 200%	[144]
	ZnO NPs + EBL	Cu	50 mg L^−1^ + 10^−8^ M	Spray + plunge	*Solanum lycopersicum*	The combination increased plant growth and biomass, photosynthetic pigments, and gaseous exchange parameters and reduced oxidative stress by lowering MDA and ROS	[145]
	ZnO NPs + EBL	Salt	50, 100 mg L^−1^ + 0.2, 0.4 μM	Seed priming	*Zea mays*	The combined treatment increased germination attributes, K^+^ content, and α-amylase activity and decreased germination time, days to 50% emergence, and Na^+^ uptake	[146]
	Ag NPs + SA	Submergence	100 mg L^−1^ + 10 µM	Foliar spray	*Oryza sativa*	Combine treatment had a better effect and induced the expression of sub1A QTL regulatory APETALA2/ETHYLENE RESPONSE FACTOR (AP2/ERF), improving stress tolerance	[147]
	Fe NPs + SA	Salt	3 mM + 1 mM	Foliar spray	*Trachyspermum ammi*	The combined treatment raised phenols, phenylalanine ammonia-lyase, and tyrosine ammonia-lyase activity, nutrients, and SA levels while decreasing Na absorption	[148]
**Metalloid + hormone**	Si NPs + MeJa	Salt	2 mM + 0.5 mM	NM	*Fragaria x ananassa*	The combined treatment upregulated stress-responsive genes and increased total soluble protein while decreasing ROS	[149]
**Mineral + carbon based**	CaO NPs + GO NPs	Drought	1, 2 PPM + 0.5, 1.5 PPM	Nutrient media	*Medicago sativa*	NPs reduced the expression of downstream genes, resulting in lower ROS, MD and reduced oxidative stress	[150]
**Mineral + hormone**	Ca NPs + ABA	Drought	100 PPM + 100 µM	Foliar spray	*Brassica napus*	Combined application improved chlorophyll, xanthophyll and the transcript level of ROS homeostasis genes, reducing drought symptoms	[151]

ABA, abscisic acid; MT, melatonin; EBL, epibrassinolide; MeJa, methyl jasmonate; SA, salicylic acid.

**Table 4:** Use of NPs as nanoremediation agents for the removal of various contaminants both from soil and water.

NMs	Pollutant	Medium	Method	Efficiency	Reference
Ag-doped TiO_2_	COD, PC	Water	Photocatalytic, biodegradation, integrated	64%, 91% 65%, 50% 62% 84%	[178]
cellulose acetate NF	Dyes	Water	biodegradation	91.3%	[179]
Fe-zeolites	nitroaromatic compounds	Water	Adsorption degradation	NM	[180]
Zero valent Fe NPs	lindane	Water	degradation	99%	[181]
TiO_2_-NPs	Anthracene	Soil	Biodegradation	37.9%/30 days	[182]
Nanocellulose	Zn, Ni, Cu, Fe	Water	Adsorption	NM	[183]
AgNPs	Methylene blue	Water	Photocatalytic	65%	[184]
Fe(Hbidc)	Sulfamethoxazole		Catalytic degradation	95%	[185]
Fe-Cu	Petroleum	Soil	Adsorption	59%	[186]
Fe NPs	Methyl orange	Water	Catalytic degradation	77%	[187]
Mesoporous Si NPs	Methylene blue	Water	Adsorption	95%	[188]
Nanoscale zero-valent iron	SeVI and AsV	Water	Adsorption	28.63 mg/g 54.75 mg/g	[189]
Fe-BDC	Tetracycline hydrochloride		Adsorption	652.0 mg/g	[190]
Cuprous(I) oxide (Cu_2_O)	Methylene blue	Water/air	Photo-catalysis	87.77%	[191]
Iron/chitosan	As(III) and Sb(III)	Water	Adsorption	108.6 and 138.8 mg/g	[192]

COD, PC: chemical oxygen demand, phenolic compounds, NM: not mentioned, Sb: antimony, Fe-BDC: iron-benzene dicarboxylate.

## Data Availability

No data was used for the research described in the article.

## References

[1] Cappelli S.L., Domeignoz-Horta L.A., Loaiza V., Laine A.-L. (2022). Plant Biodiversity Promotes Sustainable Agriculture Directly and via Belowground Effects. Trends Plant Sci..

[2] Kah M., Tufenkji N., White J.C. (2019). Nano-Enabled Strategies to Enhance Crop Nutrition and Protection. Nat. Nanotechnol..

[3] Hendriks S.L., Montgomery H., Benton T., Badiane O., Castro de la Mata G., Fanzo J., Guinto R.R., Soussana J.-F. (2022). Global Environmental Climate Change, COVID-19, and Conflict Threaten Food Security and Nutrition. BMJ.

[4] Yaddanapudi R., Mishra A.K. (2022). Compound Impact of Drought and COVID-19 on Agriculture Yield in the USA. Sci. Total Environ..

[5] Falkendal T., Otto C., Schewe J., Jägermeyr J., Konar M., Kummu M., Watkins B., Puma M.J. (2021). Grain Export Restrictions during COVID-19 Risk Food Insecurity in Many Low- and Middle-Income Countries. Nat. Food.

[6] Olofsson F., Ernfors M. (2022). Frost Killed Cover Crops Induced High Emissions of Nitrous Oxide. Sci. Total Environ..

[7] Zhu Y., Chen F., Zhao X., Yan D., Yong W., Zhao J. (2019). Cobalt(III)-Catalyzed Intermolecular Carboamination of Propiolates and Bicyclic Alkenes via Non-Annulative Redox-Neutral Coupling. Org. Lett..

[8] Zhang Y., Li Z., Ge W., Wang J., Guo X., Wang T., Li W. (2022). Assessment of the Impact of Floods on Terrestrial Plant Biodiversity. J. Clean. Prod..

[9] Kinnunen P., Guillaume J.H.A., Taka M., D’Odorico P., Siebert S., Puma M.J., Jalava M., Kummu M. (2020). Local Food Crop Production Can Fulfil Demand for Less than One-Third of the Population. Nat. Food.

[10] Guo B., Hong C., Tong W., Xu M., Huang C., Yin H., Lin Y., Fu Q. (2020). Health Risk Assessment of Heavy Metal Pollution in a Soil-Rice System: A Case Study in the Jin-Qu Basin of China. Sci. Rep..

[11] Zeeshan M., Hu Y.X., Guo X.H., Sun C.Y., Salam A., Ahmad S., Muhammad I., Nasar J., Jahan M.S., Fahad S. (2023). Physiological and Transcriptomic Study Reveal SeNPs-Mediated AsIII Stress Detoxification Mechanisms Involved Modulation of Antioxidants, Metal Transporters, and Transcription Factors in *Glycine max* L. (Merr.) Roots. Environ. Pollut..

[12] Noman M., Ahmed T., Hussain S., Niazi M.B.K., Shahid M., Song F. (2020). Biogenic Copper Nanoparticles Synthesized by Using a Copper-Resistant Strain Shigella Flexneri SNT22 Reduced the Translocation of Cadmium from Soil to Wheat Plants. J. Hazard. Mater..

[13] Salam A., Rehman M., Qi J., Khan A.R., Yang S., Zeeshan M., Ulhassan Z., Afridi M.S., Yang C., Chen N. (2024). Cobalt Stress Induces Photosynthetic and Ultrastructural Distortion by Disrupting Cellular Redox Homeostasis in Maize. Environ. Exp. Bot..

[14] Haris M., Hussain T., Mohamed H.I., Khan A., Ansari M.S., Tauseef A., Khan A.A., Akhtar N. (2023). Nanotechnology—A New Frontier of Nano-Farming in Agricultural and Food Production and Its Development. Sci. Total Environ..

[15] Kah M., Sabliov C., Wang Y., White J.C. (2023). Nanotechnology as a Foundational Tool to Combat Global Food Insecurity. One Earth.

[16] Zhou X., El-Sappah A.H., Khaskhoussi A., Huang Q., Atif A.M., Elhamid M.A.A., Ihtisham M., El-Maati M.F.A., Soaud S.A., Tahri W. (2025). Nanoparticles: A Promising Tool against Environmental Stress in Plants. Front. Plant Sci..

[17] Kim D., Kadam A., Shinde S., Saratale R.G., Patra J., Ghodake G. (2018). Recent Developments in Nanotechnology Transforming the Agricultural Sector: A Transition Replete with Opportunities. J. Sci. Food Agric..

[18] Alvar-Beltrán J., Dibari C., Ferrise R., Bartoloni N., Marta A.D. (2023). Modelling Climate Change Impacts on Crop Production in Food Insecure Regions: The Case of Niger. Eur. J. Agron..

[19] Wang Q., Dima M., Ho-Foster A., Molebatsi K., Modongo C., Zetola N.M., Shin S.S. (2022). The Association of Household Food Insecurity and HIV Infection with Common Mental Disorders among Newly Diagnosed Tuberculosis Patients in Botswana. Public Health Nutr..

[20] Wang D., Andrée B.P.J., Chamorro A.F., Spencer P.G. (2022). Transitions into and out of Food Insecurity: A Probabilistic Approach with Panel Data Evidence from 15 Countries. World Dev..

[21] Javaid M., Haleem A., Khan I.H., Suman R. (2023). Understanding the Potential Applications of Artificial Intelligence in Agriculture Sector. Adv. Agrochem.

[22] Ghosh S., Majee M., Litwack G. (2023). Chapter Fifteen—Protein l-IsoAspartyl Methyltransferase (PIMT) and Antioxidants in Plants. Vitamins and Hormones.

[23] Kumar S. (2020). Abiotic Stresses and Their Effects on Plant Growth, Yield and Nutritional Quality of Agricultural Produce. Int. J. Food Sci. Agric..

[24] Rehman M., Saeed M.S., Fan X., Salam A., Munir R., Yasin M.U., Khan A.R., Muhammad S., Ali B., Ali I. (2023). The Multifaceted Role of Jasmonic Acid in Plant Stress Mitigation: An Overview. Plants.

[25] Rasheela A.R.P., Khalid M.F., Abumaali D.A., Alatalo J.M., Ahmed T. (2024). Impact of Abiotic Stressors on Soil Microbial Communities: A Focus on Antibiotics and Their Interactions with Emerging Pollutants. Soil Syst..

[26] Zhang Y., Xu J., Li R., Ge Y., Li Y., Li R. (2023). Plants’ Response to Abiotic Stress: Mechanisms and Strategies. Int. J. Mol. Sci..

[27] Rajput P., Singh A., Agrawal S., Ghazaryan K., Rajput V.D., Movsesyan H., Mandzhieva S., Minkina T., Alexiou A. (2024). Effects of Environmental Metal and Metalloid Pollutants on Plants and Human Health: Exploring Nano-Remediation Approach. Stress Biol..

[28] Naila S., Haq Z., Abdulllah, Salam A. (2021). Allelopathic Effect of *Taraxacum officinale* L. on Germination and Physiology of Wheat. Sustainable Intensification for Agroecosystem Services and Management.

[29] Ulhassan Z., Yang S., He D., Khan A.R., Salam A., Azhar W., Muhammad S., Ali S., Hamid Y., Khan I. (2023). Seed Priming with Nano-Silica Effectively Ameliorates Chromium Toxicity in *Brassica napus*. J. Hazard. Mater..

[30] Wu X., Song H., Guan C., Zhang Z. (2020). Boron Alleviates Cadmium Toxicity in *Brassica napus* by Promoting the Chelation of Cadmium onto the Root Cell Wall Components. Sci. Total Environ..

[31] Ma Y., Dias M.C., Freitas H. (2020). Drought and Salinity Stress Responses and Microbe-Induced Tolerance in Plants. Front. Plant Sci..

[32] Hasanuzzaman M., Nahar K., Alam M., Roychowdhury R., Fujita M. (2013). Physiological, Biochemical, and Molecular Mechanisms of Heat Stress Tolerance in Plants. Int. J. Mol. Sci..

[33] Yadav S., Modi P., Dave A., Vijapura A., Patel D., Patel M., Hasanuzzaman M., Filho M.C.M.T., Fujita M., Nogueira T.A.R. (2020). Effect of Abiotic Stress on Crops. Sustainable Crop Production.

[34] Dilnawaz F., Misra A.N., Apostolova E. (2023). Involvement of Nanoparticles in Mitigating Plant’s Abiotic Stress. Plant Stress.

[35] Azhar W., Salam A., Khan A.R., Ahmad I., Gan Y. (2025). Brassinosteroids Alleviate Ethylene-Induced Copper Oxide Nanoparticle Toxicity and Ultrastructural and Stomatal Damage in Rice Seedlings. Agriculture.

[36] Tiwari P., Bose S.K., Park K.-I., Dufossé L., Fouillaud M. (2024). Plant-Microbe Interactions under the Extreme Habitats and Their Potential Applications. Microorganisms.

[37] Afridi M.S., Javed M.A., Ali S., De Medeiros F.H.V., Ali B., Salam A., Sumaira, Marc R.A., Alkhalifah D.H.M., Selim S. (2022). New Opportunities in Plant Microbiome Engineering for Increasing Agricultural Sustainability under Stressful Conditions. Front. Plant Sci..

[38] Zhang X., Li M., Yang H., Li X., Cui Z. (2018). Physiological Responses of *Suaeda glauca* and *Arabidopsis thaliana* in Phytoremediation of Heavy Metals. J. Environ. Manag..

[39] Zhang W., Wang C., Xue R., Wang L. (2019). Effects of Salinity on the Soil Microbial Community and Soil Fertility. J. Integr. Agric..

[40] Santos-Medellín C., Edwards J., Liechty Z., Nguyen B., Sundaresan V. (2017). Drought Stress Results in a Compartment-Specific Restructuring of the Rice Root-Associated Microbiomes. mBio.

[41] Sannino D. (2021). Types and Classification of Nanomaterials. Nanotechnology.

[42] Khan Y., Sadia H., Ali Shah S.Z., Khan M.N., Shah A.A., Ullah N., Ullah M.F., Bibi H., Bafakeeh O.T., Ben Khedher N. (2022). Classification, Synthetic, and Characterization Approaches to Nanoparticles, and Their Applications in Various Fields of Nanotechnology: A Review. Catalysts.

[43] Mekuye B., Abera B. (2023). Nanomaterials: An Overview of Synthesis, Classification, Characterization, and Applications. Nano Sel..

[44] Malhotra B.D., Ali M.A. (2018). Nanocomposite Materials. Nanomaterials for Biosensors.

[45] Wang D., Saleh N.B., Byro A., Zepp R., Sahle-Demessie E., Luxton T.P., Ho K.T., Burgess R.M., Flury M., White J.C. (2022). Nano-Enabled Pesticides for Sustainable Agriculture and Global Food Security. Nat. Nanotechnol..

[46] Wu H., Li Z. (2022). Nano-Enabled Agriculture: How Do Nanoparticles Cross Barriers in Plants?. Plant Commun..

[47] Sharma R., Kumar V. (2024). Nano Enabled Agriculture for Sustainable Soil. Waste Manag. Bull..

[48] Singh A., Sharma A., Singh O., Rajput V.D., Movsesyan H., Minkina T., Alexiou A., Papadakis M., Singh R.K., Singh S. (2024). In-Depth Exploration of Nanoparticles for Enhanced Nutrient Use Efficiency and Abiotic Stresses Management: Present Insights and Future Horizons. Plant Stress.

[49] Fatima F., Hashim A., Anees S. (2021). Efficacy of Nanoparticles as Nanofertilizer Production: A Review. Environ. Sci. Pollut. Res..

[50] Chen J., Wei X. (2018). Controlled-Release Fertilizers as a Means to Reduce Nitrogen Leaching and Runoff in Container-Grown Plant Production. Nitrogen in Agriculture—Updates.

[51] Tarafder C., Daizy M., Alam M.M., Ali M.R., Islam M.J., Islam R., Ahommed M.S., Aly Saad Aly M., Khan M.Z.H. (2020). Formulation of a Hybrid Nanofertilizer for Slow and Sustainable Release of Micronutrients. ACS Omega.

[52] Kumar N., Samota S.R., Venkatesh K., Tripathi S.C. (2023). Global Trends in Use of Nano-Fertilizers for Crop Production: Advantages and Constraints—A Review. Soil Tillage Res..

[53] Lowry G.V., Gregory K.B., Apte S.C., Lead J.R. (2012). Transformations of Nanomaterials in the Environment. Environ. Sci. Technol..

[54] Avila-Quezada G.D., Ingle A.P., Golińska P., Rai M. (2022). Strategic Applications of Nano-Fertilizers for Sustainable Agriculture: Benefits and Bottlenecks. Nanotechnol. Rev..

[55] El-Saadony M.T., ALmoshadak A.S., Shafi M.E., Albaqami N.M., Saad A.M., El-Tahan A.M., Desoky E.-S.M., Elnahal A.S.M., Almakas A., Abd El-Mageed T.A. (2021). Vital Roles of Sustainable Nano-Fertilizers in Improving Plant Quality and Quantity-an Updated Review. Saudi J. Biol. Sci..

[56] Esper Neto M., Britt D.W., Lara L.M., Cartwright A., dos Santos R.F., Inoue T.T., Batista M.A. (2020). Initial Development of Corn Seedlings after Seed Priming with Nanoscale Synthetic Zinc Oxide. Agronomy.

[57] Salam A., Afridi M.S., Javed M.A., Saleem A., Hafeez A., Khan A.R., Zeeshan M., Ali B., Azhar W., Sumaira (2022). Nano-Priming against Abiotic Stress: A Way Forward towards Sustainable Agriculture. Sustainability.

[58] Salam A., Khan A.R., Liu L., Yang S., Azhar W., Ulhassan Z., Zeeshan M., Wu J., Fan X., Gan Y. (2022). Seed Priming with Zinc Oxide Nanoparticles Downplayed Ultrastructural Damage and Improved Photosynthetic Apparatus in Maize under Cobalt Stress. J. Hazard. Mater..

[59] Mehmood S., Kumar N., Mansoori A., Mohan M., Kumar A., Ghorai T.K. (2024). Effect of ZnMgO_2_ Nanoparticles Used as a Nanofertilizer: Promoting the Growth Activities of Rice Seedlings. Environ. Sci. Nano.

[60] Saurabh A., Kaur M., Khan R., Guleria G., Shandilya M., Thakur S. (2024). Foliar Application of Fe_2_O_3_ Nanofertilizer on Growth and Yield of Cauliflower (*Brassica oleracea* var. *Botrytis* L.) cv. Pusa Snowball K-1. Int. J. Phytoremediation.

[61] Upadhyay P.K., Dey A., Singh V.K., Dwivedi B.S., Singh R.K., Rajanna G.A., Babu S., Rathore S.S., Shekhawat K., Rai P.K. (2024). Changes in Microbial Community Structure and Yield Responses with the Use of Nano-Fertilizers of Nitrogen and Zinc in Wheat–Maize System. Sci. Rep..

[62] Chen S., Dong C., Gao Y., Li Y., Shi Y. (2024). Effects of Nanocarbon and Nano-calcium Carbonate on Soil Enzyme Activities and Soil Microbial Community in Wheat (*Triticum aestivum* L.) Rhizosphere Soil. J. Plant Nutr. Soil Sci..

[63] Verma K.K., Song X.P., Joshi A., Rajput V.D., Singh M., Sharma A., Singh R.K., Li D.M., Arora J., Minkina T. (2022). Nanofertilizer Possibilities for Healthy Soil, Water, and Food in Future: An Overview. Front. Plant Sci..

[64] Li H., Rehman A., ur Rahman S., Li K., Yang T., Akuetteh P., Khalid M. (2024). Biosynthesized Zinc Oxide Nanoparticles Modulate the Phytoremediation Potential of *Pennisetum giganteum* and Its Rhizocompartments Associated Microbial Community Structure. J. Clean. Prod..

[65] Dinesh G.K., Karthika S., Ragul S., Sinduja M., Sathya V., Sivashankari L., Divyabharathi P., Elangovan A., Prasad S. (2023). Effect of Nano-Formulated Agrochemicals on Rhizospheric Communities in Millets.

[66] Kumar R., Dadhich A., Dhiman M., Sharma L., Sharma M.M. (2024). Stimulatory Effect of ZnO Nanoparticles as a Nanofertilizer in Seed Priming of Pearl Millet (*Pennisetum glaucum*) and Their Bioactivity Studies. S. Afr. J. Bot..

[67] Singh N., Singh M.K., Raghuvansi J., Yadav R.K., Azim Z. (2024). Green Synthesis of Nano Iron Oxide Using *Emblica officinalis* L. Fruit Extract and Its Impact on Growth, Chlorophyll Content, and Metabolic Activity of *Solanum lycopersicum* L.. J. Appl. Biol. Biotechnol..

[68] Garza-Alonso C.A., Cadenas-Pliego G., Juárez-Maldonado A., González-Fuentes J.A., Tortella G., Benavides-Mendoza A. (2023). Fe_2_O_3_ Nanoparticles Can Replace Fe-EDTA Fertilizer and Boost the Productivity and Quality of *Raphanus sativus* in a Soilless System. Sci. Hortic..

[69] Wang Q., Xu S., Zhong L., Zhao X., Wang L. (2023). Effects of Zinc Oxide Nanoparticles on Growth, Development, and Flavonoid Synthesis in Ginkgo Biloba. Int. J. Mol. Sci..

[70] Mustafa M., Azam M., Nawaz Bhatti H., Khan A., Zafar L., Rehan Abbasi A.M. (2024). Green Fabrication of Copper Nano-Fertilizer for Enhanced Crop Yield in Cowpea Cultivar: A Sustainable Approach. Biocatal. Agric. Biotechnol..

[71] Berríos D., Nahuelcura J., González F., Peña F., Cornejo P., Pérez-Navarro J., Gómez-Alonso S., Ruiz A. (2024). The Biosynthesis, Accumulation of Phenolic Compounds and Antioxidant Response in *Lactuca sativa* L. Plants Inoculated with a Biofertilizer Based on Soil Yeast and Iron Nanoparticles. Plants.

[72] Chehrehnoorani F., Rahdari P., Mostafavi Rad M., Asadi M., Kiabi S. (2024). Evaluation of the Seed Quality and Fatty Acids Composition of Groundnut (*Arachis hypogaea* L.) in Response to the Conventional and Nano Forms of Zn and Ca Based Fertilizers. Commun. Soil Sci. Plant Anal..

[73] Li W., Keller A.A. (2024). Assessing the Impacts of Cu and Mo Engineered Nanomaterials on Crop Plant Growth Using a Targeted Proteomics Approach. ACS Agric. Sci. Technol..

[74] Motlhalamme T., Mohamed H., Kaningini A.G., More G.K., Thema F.T., Mohale K.C., Maaza M. (2023). Bio-Synthesized Calcium Carbonate (CaCO_3_) Nanoparticles: Their Anti-Fungal Properties and Application as Nanofertilizer on *Lycopersicon esculentum* Growth and Gas Exchange Measurements. Plant Nano Biol..

[75] Noruzi M., Hadian P., Soleimanpour L., Ma’mani L., Shahbazi K. (2023). Hydroxyapatite Nanoparticles: An Alternative to Conventional Phosphorus Fertilizers in Acidic Culture Media. Chem. Biol. Technol. Agric..

[76] Metwally R.A., Abdelhameed R.E. (2024). Co-Application of Arbuscular Mycorrhizal Fungi and Nano-ZnFe_2_O_4_ Improves Primary Metabolites, Enzymes and NPK Status of Pea (*Pisum sativum* L.) Plants. J. Plant Nutr..

[77] Wu H., Wan X., Niu J., Xu H., Zhang Y., Xue X., Li Y., Li Q., Lu T., Yu H. (2023). Enhancing Lettuce Yield via Cu/Fe-Layered Double Hydroxide Nanoparticles Spraying. J. Nanobiotechnol..

[78] Rojas-Velázquez Á.N., Guillén-Castillo O.I., Alcalá-Jauregui J.A., Loredo-Osti C., Ramírez-Tobías H.M., Romero-Méndez M.J., Méndez-Cortés H., Hernández-Montoya A. (2023). Effect of a Nitrogenous Nanocomposite on Leaching and N Content in Lettuce in Soil Columns. Discov. Nano.

[79] Ranjani S., Hemalatha S. (2023). Influence of Polyherbal Nanoformulation on Plant Growth and Biochemical Constituents in Legume Seedlings. J. Plant Growth Regul..

[80] Gil-Díaz M., García-Gonzalo P., Mancho C., Hernández L.E., Alonso J., Lobo M.C. (2022). Response of Spinach Plants to Different Doses of Two Commercial Nanofertilizers. Sci. Hortic..

[81] Moloudzadeh R., Fathi S., Yari F., Najafian S., Seyedi A. (2024). The Potential of Coated Iron Nanoparticles for Modulating of Negative Effects of Salinity Stress in Ajowan. Hortic. Environ. Biotechnol..

[82] Ayyaz A., Fang R., Ma J., Hannan F., Huang Q., Athar H.-R., Sun Y., Javed M., Ali S., Zhou W. (2022). Calcium Nanoparticles (Ca-NPs) Improve Drought Stress Tolerance in *Brassica napus* by Modulating the Photosystem II, Nutrient Acquisition and Antioxidant Performance. NanoImpact.

[83] Ali S., Bai Y., Zhang J., Zada S., Khan N., Hu Z., Tang Y. (2024). Discovering Nature’s Shield: Metabolomic Insights into Green Zinc Oxide Nanoparticles Safeguarding *Brassica parachinensis* L. from Cadmium Stress. Plant Physiol. Biochem..

[84] Nawaz H., Ahmad K.S., Aslam I., Mahmood A., Mehmood A., Khan A., Jamil M., Ameer A., Khan M.F., Hussain S. (2024). Modulations in Physiological and Biochemical Attributes of *Citrus limon* by Selenium Nanoparticles (SeNPs) under Salinity Stress. Biocatal. Agric. Biotechnol..

[85] Zafar S., Hasnain Z., Danish S., Battaglia M.L., Fahad S., Ansari M.J., Alharbi S.A. (2024). Modulations of Wheat Growth by Selenium Nanoparticles under Salinity Stress. BMC Plant Biol..

[86] Shang H., Li C., Cai Z., Hao Y., Cao Y., Jia W., Han L., White J.C., Ma C., Xing B. (2024). Biosynthesized Selenium Nanoparticles as an Effective Tool to Combat Soil Metal Stresses in Rice (*Oryza sativa* L.). ACS Nano.

[87] Chen M., Jiao S., Xie L., Geng X., Qi S., Fan J., Cheng S., Shi J., Cao X. (2024). Integrated Physiological, Transcriptomic, and Metabolomic Analyses of Drought Stress Alleviation in *Ehretia macrophylla* Wall. Seedlings by SiO_2_ NPs (Silica Nanoparticles). Front. Plant Sci..

[88] Sulaiman, Ahmad A., Noor Hassim M.F. (2024). Effects of Silica Nanoparticles on Morpho-Histological and Antioxidant Activities of Rice Seedlings under Drought Stress. S. Afr. J. Bot..

[89] Alomrani S.O., Kaleem M., Aslam M., Habib F., Jamal A., Waseem M., Javed T., Wahid A. (2024). Copper Nanoparticles Alleviate Cadmium Stress in *Solanum melongena* through Endogenous Melatonin and Regulation of Some Physiochemical Attributes. Sci. Hortic..

[90] Kumar D., Mariyam S., Gupta K.J., Thiruvengadam M., Sampatrao Ghodake G., Xing B., Seth C.S. (2024). Comparative Investigation on Chemical and Green Synthesized Titanium Dioxide Nanoparticles against Chromium (VI) Stress Eliciting Differential Physiological, Biochemical, and Cellular Attributes in *Helianthus annuus* L.. Sci. Total Environ..

[91] Ullah Z., Haq S.I.U., Ullah A., Asghar M.A., Seleiman M.F., Saleem K., Zeng F., Sama N.U., Kamran K., Ahmad S. (2025). Effect of Green Synthesized Silver Nanoparticles on Growth and Physiological Responses of Pearl Millet under Salinity Stress. Environ. Dev. Sustain..

[92] Zhuang Q., Zhang Y., Liu Q., Sun Y., Sharma S., Tang S., Dhankher O.P., Yuan H. (2024). Effects of Sulfur Nanoparticles on Rhizosphere Microbial Community Changes in Oilseed Rape Plantation Soil under Mercury Stress. Int. J. Phytoremediation.

[93] Tahir K., Haroon U., Akbar M., Elahi M., Quraishi U.M. (2024). Tetragonal Crystalline MnO Nanoparticles Alleviate Pb Stress in Wheat by Modulating Antioxidant Enzymes in Leaves. Physiol. Mol. Biol. Plants.

[94] Mohamed H.I., Ullah I., Toor M.D., Tanveer N.A., Din M.M.U., Basit A., Sultan Y., Muhammad M., Rehman M.U. (2025). Heavy Metals Toxicity in Plants: Understanding Mechanisms and Developing Coping Strategies for Remediation: A Review. Bioresour. Bioprocess..

[95] Huang Q., Ayyaz A., Farooq M.A., Zhang K., Chen W., Hannan F., Sun Y., Shahzad K., Ali B., Zhou W. (2024). Silicon Dioxide Nanoparticles Enhance Plant Growth, Photosynthetic Performance, and Antioxidants Defence Machinery through Suppressing Chromium Uptake in *Brassica napus* L.. Environ. Pollut..

[96] Rehman M., Salam A., Ali B., Ahmad I., Javaid M.H., Haider Z., Munir R., Yasin M.U., Ali I., Yang C. (2025). Titanium Dioxide Nanoparticles Seed Priming as a Remedy for Nickel-Induced Stress in Maize through Antioxidant Enhancement and Ultrastructural Optimization. J. Environ. Manag..

[97] Prakash V., Rai P., Sharma N.C., Singh V.P., Tripathi D.K., Sharma S., Sahi S. (2022). Application of Zinc Oxide Nanoparticles as Fertilizer Boosts Growth in Rice Plant and Alleviates Chromium Stress by Regulating Genes Involved in Oxidative Stress. Chemosphere.

[98] Basit F., Shahid M., Abbas S., Naqqash T., Akram M.S., Tahir M., Azeem M., Cai Y., Jia S., Hu J. (2023). Protective Role of ZnO Nanoparticles in Soybean Seedlings Growth and Stress Management under Cr-Enriched Conditions. Plant Growth Regul..

[99] Ahmed T., Masood H.A., Noman M., AL-Huqail A.A., Alghanem S.M., Khan M.M., Muhammad S., Manzoor N., Rizwan M., Qi X. (2023). Biogenic Silicon Nanoparticles Mitigate Cadmium (Cd) Toxicity in Rapeseed (*Brassica napus* L.) by Modulating the Cellular Oxidative Stress Metabolism and Reducing Cd Translocation. J. Hazard. Mater..

[100] Bashir S.S., Hussain A., Hussain S.J., Wani O.A., Zahid Nabi S., Dar N.A., Baloch F.S., Mansoor S. (2021). Plant Drought Stress Tolerance: Understanding Its Physiological, Biochemical and Molecular Mechanisms. Biotechnol. Biotechnol. Equip..

[101] Van Nguyen D., Nguyen H.M., Le N.T., Nguyen K.H., Nguyen H.T., Le H.M., Nguyen A.T., Dinh N.T.T., Hoang S.A., Van Ha C. (2022). Copper Nanoparticle Application Enhances Plant Growth and Grain Yield in Maize Under Drought Stress Conditions. J. Plant Growth Regul..

[102] Mustafa H., Ilyas N., Akhtar N., Raja N.I., Zainab T., Shah T., Ahmad A., Ahmad P. (2021). Biosynthesis and Characterization of Titanium Dioxide Nanoparticles and Its Effects along with Calcium Phosphate on Physicochemical Attributes of Wheat under Drought Stress. Ecotoxicol. Environ. Saf..

[103] Zeeshan M., Wang X., Salam A., Wu H., Li S., Zhu S., Chang J., Chen X., Zhang Z., Zhang P. (2024). Selenium Nanoparticles Boost the Drought Stress Response of Soybean by Enhancing Pigment Accumulation, Oxidative Stress Management and Ultrastructural Integrity. Agronomy.

[104] Boora R., Rani N., Kumari S., Goel S., Arya A., Grewal S. (2024). Exploring the Role of Green Synthesized Cerium Nanoparticles in Enhancing Wheat’s Drought Tolerance: A Comprehensive Study of Biochemical Parameters and Gene Expression. Cereal Res. Commun..

[105] Daler S. (2024). Improving Grapevine (*Vitis vinifera* L., cv. Superior Seedless) Drought Tolerance with Cerium Oxide Nanoparticles: Agronomic and Molecular Insights. Sci. Hortic..

[106] Raza M.A.S., Amin J., Valipour M., Iqbal R., Aslam M.U., Zulfiqar B., Muhammad F., Ibrahim M.A., Al-Ghamdi A.A., Elshikh M.S. (2024). Cu-Nanoparticles Enhance the Sustainable Growth and Yield of Drought-Subjected Wheat through Physiological Progress. Sci. Rep..

[107] Javan M., Selahvarzi Y., Sayyad-Amin P., Rastegar S. (2024). Potential Application of TiO_2_ Nanoparticles to Improve the Nutritional Quality of Strawberry cv. Camarosa under Drought Stress. Sci. Hortic..

[108] Chhabra R. (2021). Nature and Origin of Salts, Classification, Area and Distribution of Salt-Affected Soils. Salt-Affected Soils and Marginal Waters.

[109] Feng D., Gao Q., Liu J., Tang J., Hua Z., Sun X. (2023). Categories of Exogenous Substances and Their Effect on Alleviation of Plant Salt Stress. Eur. J. Agron..

[110] Hoang T., Tran T., Nguyen T., Williams B., Wurm P., Bellairs S., Mundree S. (2016). Improvement of Salinity Stress Tolerance in Rice: Challenges and Opportunities. Agronomy.

[111] Singh A., Rajput V.D., Agrawal S., Ghazaryan K., Minkina T., Al Tawaha A.R.M., Chauhan A., Mandzhieva S.S., Singh R.K., Papadakis M. (2024). Nanoparticles Mediated Salt Stress Resilience: A Holistic Exploration of Physiological, Biochemical, and Nano-Omics Approaches. Rev. Environ. Contam. Toxicol..

[112] Fouda H.M., Saied E., Abdelmouty E.S., Osman M.S. (2024). Ameliorative Role of Copper Nanoparticle in Alleviating Salt-Induced Oxidative Stress in Fenugreek (*Trigonella foenum-graecum* L.) Plants. Biocatal. Agric. Biotechnol..

[113] Amir M., Raheem A., Yadav P., Kumar V., Tewari R.K., Jalil S.U., Danish M., Ansari M.I. (2024). Phytofabricated Gold Nanoparticles as Modulators of Salt Stress Responses in Spinach: Implications for Redox Homeostasis, Biochemical and Physiological Adaptation. Front. Plant Sci..

[114] Khalid M.F., Jawaid M.Z., Nawaz M., Shakoor R.A., Ahmed T. (2024). Employing Titanium Dioxide Nanoparticles as Biostimulant against Salinity: Improving Antioxidative Defense and Reactive Oxygen Species Balancing in Eggplant Seedlings. Antioxidants.

[115] Song Y., Zheng C., Basnet R., Li S., Chen J., Jiang M. (2022). Astaxanthin Synthesized Gold Nanoparticles Enhance Salt Stress Tolerance in Rice by Enhancing Tetrapyrrole Biosynthesis and Scavenging Reactive Oxygen Species In Vitro. Plant Stress.

[116] Adnan M., Mahmood F., Zhao Z., Khaliq H., Usman M., Muhammad T., Ashraf G.A. (2025). Effect of the Foliar Application of Biogenic-ZnO Nanoparticles on Physio-Chemical Analysis of Chilli (*Capsicum annum* L.) in a Salt Stress Environment. Environ. Sci. Adv..

[117] Gao J., Ding Y., Liu Y., He Y., Zhao D., Su X., Gao L., Song K., He X. (2025). Effects of Seed Priming with La_2_O_3_ Nanoparticles on Seed Vigor of Alfalfa (*Medicago sativa*) under Salt Stress. S. Afr. J. Bot..

[118] Khatoon S., Mahajan M., Kumari S., Iqbal N., Wahid I., Khan M.I.R. (2025). Green-Synthesized Gold Nanoparticles Induce Adaptation in Photosynthetic Responses, Sugar and Nitrogen Metabolism, and Seed Yield of Salt-Stressed Mustard Plants. Clean Technol. Environ. Policy.

[119] Chen S., Long L., Sun X., Parsons D., Zhou Z. (2025). Responsive Root Traits and Mitigating Strategies for Wheat Production under Single or Combined Abiotic Stress. Eur. J. Agron..

[120] Khan I., Awan S.A., Rizwan M., Huizhi W., Ulhassan Z., Xie W. (2024). Silicon Nanoparticles Improved the Osmolyte Production, Antioxidant Defense System, and Phytohormone Regulation in *Elymus sibiricus* (L.) under Drought and Salt Stress. Environ. Sci. Pollut. Res..

[121] Al–Mayahi A.M.W. (2023). Combined Efficiency of Iron Nanoparticles (IONPs) and Salicylic Acid (SA) on In Vitro Propagation of Date Palm (*Phoenix dactylifera* L.) under Combined Drought and Salinity. S. Afr. J. Bot..

[122] Adrees M., Khan Z.S., Ali S., Hafeez M., Khalid S., ur Rehman M.Z., Hussain A., Hussain K., Shahid Chatha S.A., Rizwan M. (2020). Simultaneous Mitigation of Cadmium and Drought Stress in Wheat by Soil Application of Iron Nanoparticles. Chemosphere.

[123] Ahmed T., Noman M., Manzoor N., Shahid M., Abdullah M., Ali L., Wang G., Hashem A., Al-Arjani A.-B.F., Alqarawi A.A. (2021). Nanoparticle-Based Amelioration of Drought Stress and Cadmium Toxicity in Rice via Triggering the Stress Responsive Genetic Mechanisms and Nutrient Acquisition. Ecotoxicol. Environ. Saf..

[124] El-Saadony M.T., Desoky E.-S.M., Saad A.M., Eid R.S.M., Selem E., Elrys A.S. (2021). Biological Silicon Nanoparticles Improve *Phaseolus vulgaris* L. Yield and Minimize Its Contaminant Contents on a Heavy Metals-Contaminated Saline Soil. J. Environ. Sci..

[125] Toaiema W.I.M., Mustafa S.S.S. (2024). Silicon Nanoparticle Application on Thymus Serpyllum Under Drought and Salinity Stress In Vitro. Bionanoscience.

[126] Omar A.A., Heikal Y.M., Zayed E.M., Shamseldin S.A.M., Salama Y.E., Amer K.E., Basuoni M.M., Abd Ellatif S., Mohamed A.H. (2023). Conferring of Drought and Heat Stress Tolerance in Wheat (*Triticum aestivum* L.) Genotypes and Their Response to Selenium Nanoparticles Application. Nanomaterials.

[127] El-Saadony M.T., Saad A.M., Najjar A.A., Alzahrani S.O., Alkhatib F.M., Shafi M.E., Selem E., Desoky E.-S.M., Fouda S.E.E., El-Tahan A.M. (2021). The Use of Biological Selenium Nanoparticles to Suppress *Triticum aestivum* L. Crown and Root Rot Diseases Induced by Fusarium Species and Improve Yield under Drought and Heat Stress. Saudi J. Biol. Sci..

[128] Dinler B.S., Cetinkaya H., Koc F.N., Gül V., Sefaoğlu F. (2024). Effects of Titanium Dioxide Nanoparticles against Salt and Heat Stress in Safflower Cultivars. Acta Bot. Bras..

[129] Zahedi S.M., Abdelrahman M., Hosseini M.S., Hoveizeh N.F., Tran L.-S.P. (2019). Alleviation of the Effect of Salinity on Growth and Yield of Strawberry by Foliar Spray of Selenium-Nanoparticles. Environ. Pollut..

[130] Bhat J.A., Faizan M., Bhat M.A., Huang F., Yu D., Ahmad A., Bajguz A., Ahmad P. (2022). Defense Interplay of the Zinc-Oxide Nanoparticles and Melatonin in Alleviating the Arsenic Stress in Soybean (*Glycine max* L.). Chemosphere.

[131] Faiz S., Shah A.A., Naveed N.H., Nijabat A., Yasin N.A., Batool A.I., Ali H.M., Javed T., Simon P.W., Ali A. (2022). Synergistic Application of Silver Nanoparticles and Indole Acetic Acid Alleviate Cadmium Induced Stress and Improve Growth of *Daucus carota* L.. Chemosphere.

[132] Sheikhalipour M., Gohari G., Esmaielpour B., Panahirad S., Milani M.H., Kulak M., Janda T. (2023). Melatonin and TiO_2_ NPs Application-Induced Changes in Growth, Photosynthesis, Antioxidant Enzymes Activities and Secondary Metabolites in Stevia (*Stevia rebaudiana* Bertoni) Under Drought Stress Conditions. J. Plant Growth Regul..

[133] Soliman M.H., Alghanem S.M.S., Alsudays I.M., Alaklabi A., Alharbi B.M., Al-Amrah H., Azab E., Alnusairi G.S.H. (2024). Co-Application of Titanium Nanoparticles and Melatonin Effectively Lowered Chromium Toxicity in Lemon Balm (*Melissa officinalis* L.) through Modifying Biochemical Characteristics. Environ. Sci. Pollut. Res..

[134] Guardiola-Márquez C.E., García-Sánchez C.V., Sánchez-Arellano Ó.A., Bojorquez-Rodríguez E.M., Jacobo-Velázquez D.A. (2023). Biofortification of Broccoli Microgreens (*Brassica oleracea* Var. *italica*) with Glucosinolates, Zinc, and Iron through the Combined Application of Bio- and Nanofertilizers. Foods.

[135] Faraji J., Sepehri A. (2020). Exogenous Nitric Oxide Improves the Protective Effects of TiO_2_ Nanoparticles on Growth, Antioxidant System, and Photosynthetic Performance of Wheat Seedlings Under Drought Stress. J. Soil Sci. Plant Nutr..

[136] Raliya R., Biswas P., Tarafdar J.C. (2015). TiO_2_ Nanoparticle Biosynthesis and Its Physiological Effect on Mung Bean (*Vigna radiata* L.). Biotechnol. Rep..

[137] Soliman M.H., Alnusairi G.S.H., Khan A.A., Alnusaire T.S., Fakhr M.A., Abdulmajeed A.M., Aldesuquy H.S., Yahya M., Najeeb U. (2023). Biochar and Selenium Nanoparticles Induce Water Transporter Genes for Sustaining Carbon Assimilation and Grain Production in Salt-Stressed Wheat. J. Plant Growth Regul..

[138] Ciccolini V., Pellegrino E., Coccina A., Fiaschi A.I., Cerretani D., Sgherri C., Quartacci M.F., Ercoli L. (2017). Biofortification with Iron and Zinc Improves Nutritional and Nutraceutical Properties of Common Wheat Flour and Bread. J. Agric. Food Chem..

[139] Bashir A., ur Rehman M.Z., Hussaini K.M., Adrees M., Qayyum M.F., Sayal A.U., Rizwan M., Ali S., Alsahli A.A., Alyemeni M.N. (2021). Combined Use of Zinc Nanoparticles and Co-Composted Biochar Enhanced Wheat Growth and Decreased Cd Concentration in Grains under Cd and Drought Stress: A Field Study. Environ. Technol. Innov..

[140] Azmat A., Tanveer Y., Yasmin H., Hassan M.N., Shahzad A., Reddy M., Ahmad A. (2022). Coactive Role of Zinc Oxide Nanoparticles and Plant Growth Promoting Rhizobacteria for Mitigation of Synchronized Effects of Heat and Drought Stress in Wheat Plants. Chemosphere.

[141] Shah A.A., Aslam S., Akbar M., Ahmad A., Khan W.U., Yasin N.A., Ali B., Rizwan M., Ali S. (2021). Combined Effect of *Bacillus fortis* IAGS 223 and Zinc Oxide Nanoparticles to Alleviate Cadmium Phytotoxicity in *Cucumis melo*. Plant Physiol. Biochem..

[142] Liu A., Xiao W., Lai W., Wang J., Li X., Yu H., Zha Y. (2024). Potential Application of Selenium and Copper Nanoparticles in Improving Growth, Quality, and Physiological Characteristics of Strawberry under Drought Stress. Agriculture.

[143] Bozaba T.O., Kuru İ.S. (2024). The Effect of the Combined Application of Elicitors to Salvia Virgata Jacq. under Salinity Stress on Physiological and Antioxidant Defense. BMC Plant Biol..

[144] Labulo A.H., David O.A., Terna A.D., Omotosho T.P., Tanko N.S., Hassan I., Oluwole B.R., Odebode A. (2024). Modulation of Physiological and Biochemical Activities of Eugenia Uniflora by Green-Synthesized Silver Nanoparticle and Melatonin under Drought Stress. Plant Biotechnol. Rep..

[145] Faizan M., Bhat J.A., Noureldeen A., Ahmad P., Yu F. (2021). Zinc Oxide Nanoparticles and 24-Epibrassinolide Alleviates Cu Toxicity in Tomato by Regulating ROS Scavenging, Stomatal Movement and Photosynthesis. Ecotoxicol. Environ. Saf..

[146] Alhammad B.A., Ahmad A., Seleiman M.F., Tola E. (2023). Seed Priming with Nanoparticles and 24-Epibrassinolide Improved Seed Germination and Enzymatic Performance of *Zea mays* L. in Salt-Stressed Soil. Plants.

[147] Saha I., Hasanuzzaman M., Dolui D., Sikdar D., Debnath S.C., Adak M.K. (2021). Silver-Nanoparticle and Abscisic Acid Modulate Sub1A Quantitative Trait Loci Functioning towards Submergence Tolerance in Rice (*Oryza sativa* L.). Environ. Exp. Bot..

[148] Abdoli S., Ghassemi-Golezani K. (2025). Foliar Treatments of Salicylic Acid and Iron Nanoparticles Enhanced Antioxidant Potential and Essential Oil Production of Ajowan under Salt Stress. Plant Biosyst.-Int. J. Deal. All Asp. Plant Biol..

[149] Moradi P., Vafaee Y., Mozafari A.A., Tahir N.A. (2022). Silicon Nanoparticles and Methyl Jasmonate Improve Physiological Response and Increase Expression of Stress-Related Genes in Strawberry Cv. Paros Under Salinity Stress. Silicon.

[150] Yazicilar B., Nadaroğlu H., Alayli A., Nadar M., Gedikli S., BezirĞanoğlu İ. (2024). Mitigation of Drought Stress Effects on Alfalfa (*Medicago sativa* L.) Callus through CaO Nanoparticles and Graphene Oxide in Tissue Culture Conditions. Plant Cell Tissue Organ Cult. (PCTOC).

[151] Ayyaz A., Zhou Y., Batool I., Hannan F., Huang Q., Zhang K., Shahzad K., Sun Y., Farooq M.A., Zhou W. (2024). Calcium Nanoparticles and Abscisic Acid Improve Drought Tolerance, Mineral Nutrients Uptake and Inhibitor-Mediated Photosystem II Performance in *Brassica napus*. J. Plant Growth Regul..

[152] Muddineti O.S., Ghosh B., Biswas S. (2015). Current Trends in Using Polymer Coated Gold Nanoparticles for Cancer Therapy. Int. J. Pharm..

[153] Kralj S., Rojnik M., Romih R., Jagodič M., Kos J., Makovec D. (2012). Effect of Surface Charge on the Cellular Uptake of Fluorescent Magnetic Nanoparticles. J. Nanoparticle Res..

[154] Mansouri E., Mesbahi A., Hamishehkar H., Montazersaheb S., Hosseini V., Rajabpour S. (2023). The Effect of Nanoparticle Coating on Biological, Chemical and Biophysical Parameters Influencing Radiosensitization in Nanoparticle-Aided Radiation Therapy. BMC Chem..

[155] Soni S., Singh K.M., Jha A.B., Dubey R.S., Sharma P. (2026). Size-Based Dynamics of Nanoparticles in Plant Growth and Environmental Stress Tolerance: Potential Benefits and Hazards. Environ. Sci. Nano.

[156] Kim S.H., Bae S., Sung Y.W., Hwang Y.S. (2024). Effects of Particle Size on Toxicity, Bioaccumulation, and Translocation of Zinc Oxide Nanoparticles to Bok Choy (*Brassica chinensis* L.) in Garden Soil. Ecotoxicol. Environ. Saf..

[157] Avramescu M.-L., Chénier M., Beauchemin S., Rasmussen P. (2022). Dissolution Behaviour of Metal-Oxide Nanomaterials in Various Biological Media. Nanomaterials.

[158] Huang B., Chen F., Shen Y., Qian K., Wang Y., Sun C., Zhao X., Cui B., Gao F., Zeng Z. (2018). Advances in Targeted Pesticides with Environmentally Responsive Controlled Release by Nanotechnology. Nanomaterials.

[159] Kumari A., Gupta A.K., Sharma S., Jadon V.S., Sharma V., Chun S.C., Sivanesan I. (2024). Nanoparticles as a Tool for Alleviating Plant Stress: Mechanisms, Implications, and Challenges. Plants.

[160] Meel S., Saharan B.S. (2024). Enhancing Crop Resilience towards Drought: By Integrating Nanotechnology, Microbiomes, and Growth-Promoting Rhizobacteria. Discov. Agric..

[161] Zhao Y., Tang Y., Hu L., Xu J., Zhang X., Dou X., Zhang S., Huang L., Wang X. (2025). Growth and Physiology Effects of Seed Priming and Foliar Application of ZnO Nanoparticles on *Hibiscus syriacus* L.. Sci. Rep..

[162] Nehra M., Dilbaghi N., Singhal N.K., Hassan A.A., Kim K.-H., Kumar S. (2019). Metal Organic Frameworks MIL-100(Fe) as an Efficient Adsorptive Material for Phosphate Management. Environ. Res..

[163] Mahmoud M.E., Amira M.F., Seleim S.M., Mohamed A.K. (2020). Amino-Decorated Magnetic Metal-Organic Framework as a Potential Novel Platform for Selective Removal of Chromium (Vl), Cadmium (II) and Lead (II). J. Hazard. Mater..

[164] Baazaoui N., Bellili K., Messaoud M., Elleuch L., Elleuch R., Labidi S., Aounallah K., Maazoun A., Salhi R., Shati A.A. (2023). Bio-Nano-Remediation of Olive Oil Mill Wastewater Using Silicon Dioxide Nanoparticles for Its Potential Use as Biofertilizer for Young Olive Plants. Silicon.

[165] Ghazaryan K., Agrawal S., Margaryan G., Harutyunyan A., Rajput P., Movsesyan H., Rajput V.D., Singh R.K., Minkina T., Elshikh M.S. (2024). Soil Pollution: An Agricultural and Environmental Problem with Nanotechnological Remediation Opportunities and Challenges. Discov. Sustain..

[166] Yang Q., Cui P., Liu C., Fang G., Dang F., Wang P., Wang S., Wang Y. (2024). Core–Shell CoN@Co Ultra-Stable Nanoparticles on Biochar for Contamination Remediation in Water and Soil. Carbon Res..

[167] Xie L., Ma Q., Chen Q., Liu Y., Guo P., Zhang J., Duan G., Lin A., Zhang T., Li S. (2025). Efficient Remediation of Different Concentrations of Cr-Contaminated Soils by Nano Zero-Valent Iron Modified with Carboxymethyl Cellulose and Biochar. J. Environ. Sci..

[168] Vojoudi H., Badiei A., Bahar S., Mohammadi Ziarani G., Faridbod F., Ganjali M.R. (2017). A New Nano-Sorbent for Fast and Efficient Removal of Heavy Metals from Aqueous Solutions Based on Modification of Magnetic Mesoporous Silica Nanospheres. J. Magn. Magn. Mater..

[169] Kamath V., Chandra P., Jeppu G.P. (2020). Comparative Study of Using Five Different Leaf Extracts in the Green Synthesis of Iron Oxide Nanoparticles for Removal of Arsenic from Water. Int. J. Phytoremediation.

[170] Sithara N.V., Bharathi D., Lee J., Mythili R., Devanesan S., AlSalhi M.S. (2024). Synthesis of Iron Oxide Nanoparticles Using Orange Fruit Peel Extract for Efficient Remediation of Dye Pollutant in Wastewater. Environ. Geochem. Health.

[171] Marimuthu G., Priyadharsini C.I., Prabhu S., Viji A., Vignesh S., AlSalhi M.S., Lee J., Palanisamy G. (2024). Silver-Decorated SrTiO_3_ Nanoparticles for High-Performance Supercapacitors and Effective Remediation of Hazardous Pollutants. Environ. Geochem. Health.

[172] Matos M.P.S.R., Correia A.A.S., Rasteiro M.G. (2017). Application of Carbon Nanotubes to Immobilize Heavy Metals in Contaminated Soils. J. Nanoparticle Res..

[173] Jiang D., Zeng G., Huang D., Chen M., Zhang C., Huang C., Wan J. (2018). Remediation of Contaminated Soils by Enhanced Nanoscale Zero Valent Iron. Environ. Res..

[174] Baragaño D., Forján R., Welte L., Gallego J.L.R. (2020). Nanoremediation of As and Metals Polluted Soils by Means of Graphene Oxide Nanoparticles. Sci. Rep..

[175] Arenas-Lago D., Rodríguez-Seijo A., Lago-Vila M., Couce L.A., Vega F.A. (2016). Using Ca_3_(PO_4_)_2_ Nanoparticles to Reduce Metal Mobility in Shooting Range Soils. Sci. Total Environ..

[176] Ding L., Li J., Liu W., Zuo Q., Liang S. (2017). Influence of Nano-Hydroxyapatite on the Metal Bioavailability, Plant Metal Accumulation and Root Exudates of Ryegrass for Phytoremediation in Lead-Polluted Soil. Int. J. Environ. Res. Public Health.

[177] Joseph J., Iftekhar S., Srivastava V., Fallah Z., Zare E.N., Sillanpää M. (2021). Iron-Based Metal-Organic Framework: Synthesis, Structure and Current Technologies for Water Reclamation with Deep Insight into Framework Integrity. Chemosphere.

[178] Messaoud M., Salhi R., Baazaoui N., Hammami S.B.M., Ezzine A., Mosbeh R., Elleuch R., Labidi S., Ounallah K., Maazoun A. (2024). Bio-Nanoremediation of Olive Oil Mill Wastewater by *Alternaria alternata* Fungi Coupled with Ag-Doped TiO_2_ Nanoparticles and Its Use as Biofertilizer for Cereal Crops. EuroMediterr. J. Environ. Integr..

[179] Erkoç E., Tüzün İ., Korkmaz F., San Keskin N.O., Koçberber Kiliç N. (2024). Nanoremediation of Toxic Dyes Using a Bacterial Consortium Immobilized on Cellulose Acetate Nanofiber Mats. Polym. Eng. Sci..

[180] Gawel A., Sühnholz S., Georgi A., Kopinke F.-D., Mackenzie K. (2023). Fe-Zeolites for the Adsorption and Oxidative Degradation of Nitroaromatic Compounds in Water. J. Hazard. Mater..

[181] Ningthoujam R., Sahoo B., Ghosh P., Shivani A., Ganguli P., Chaudhuri S. (2023). Green Production of Zero-Valent Iron Nanoparticles Using Pomegranate Peel Extracts and Its Use in Lindane Degradation. Nanotechnol. Environ. Eng..

[182] Chakravarty P., Deka H., Chowdhury D. (2023). Anthracene Removal Potential of Green Synthesized Titanium Dioxide Nanoparticles (TiO_2_-NPs) and *Alcaligenes faecalis* HP8 from Contaminated Soil. Chemosphere.

[183] Guidi P., Bernardeschi M., Palumbo M., Buttino I., Vitiello V., Scarcelli V., Chiaretti G., Fiorati A., Pellegrini D., Pontorno L. (2023). Eco-Friendly Engineered Nanomaterials Coupled with Filtering Fine-Mesh Net as a Promising Tool to Remediate Contaminated Freshwater Sludges: An Ecotoxicity Investigation. Nanomaterials.

[184] Filho A.C.D., de Jesus Soares J., Carriço M.R.S., Viçozi G.P., Flores W.H., Denardin C.C., Roehrs R., Denardin E.L.G. (2022). Green Synthesis Silver Nanoparticles *Bougainvillea glabra* Choisy/LED Light with High Catalytic Activity in the Removal of Methylene Blue Aqueous Solution. Environ. Sci. Pollut. Res..

[185] Pu M., Wan J., Zhang F., Brusseau M.L., Ye D., Niu J. (2021). Insight into Degradation Mechanism of Sulfamethoxazole by Metal-Organic Framework Derived Novel Magnetic Fe@C Composite Activated Persulfate. J. Hazard. Mater..

[186] Vu K.A., Mulligan C.N. (2023). Remediation of Oil-Contaminated Soil Using Fe/Cu Nanoparticles and Biosurfactants. Environ. Technol..

[187] Shaker Ardakani L., Alimardani V., Tamaddon A.M., Amani A.M., Taghizadeh S. (2021). Green Synthesis of Iron-Based Nanoparticles Using *Chlorophytum comosum* Leaf Extract: Methyl Orange Dye Degradation and Antimicrobial Properties. Heliyon.

[188] Usgodaarachchi L., Thambiliyagodage C., Wijesekera R., Bakker M.G. (2021). Synthesis of Mesoporous Silica Nanoparticles Derived from Rice Husk and Surface-Controlled Amine Functionalization for Efficient Adsorption of Methylene Blue from Aqueous Solution. Curr. Res. Green Sustain. Chem..

[189] Suazo-Hernández J., Manquián-Cerda K., de la Luz Mora M., Molina-Roco M., Angélica Rubio M., Sarkar B., Bolan N., Arancibia-Miranda N. (2021). Efficient and Selective Removal of SeVI and AsV Mixed Contaminants from Aqueous Media by Montmorillonite-Nanoscale Zero Valent Iron Nanocomposite. J. Hazard. Mater..

[190] Jung K.-W., Kim J.-H., Choi J.-W. (2020). Synthesis of Magnetic Porous Carbon Composite Derived from Metal-Organic Framework Using Recovered Terephthalic Acid from Polyethylene Terephthalate (PET) Waste Bottles as Organic Ligand and Its Potential as Adsorbent for Antibiotic Tetracycline Hydrochloride. Compos. B Eng..

[191] Muthukumaran M., Dhinagaran G., Venkatachalam K., Sagadevan S., Gunasekaran S., Podder J., Mohammad F., Shahid M.M., Oh W.C. (2020). Green Synthesis of Cuprous Oxide Nanoparticles for Environmental Remediation and Enhanced Visible-Light Photocatalytic Activity. Optik.

[192] Zeng J., Qi P., Shi J., Pichler T., Wang F., Wang Y., Sui K. (2020). Chitosan Functionalized Iron Nanosheet for Enhanced Removal of As(III) and Sb(III): Synergistic Effect and Mechanism. Chem. Eng. J..

[193] Ijaz S., Iqbal J., Abbasi B.A., Ullah Z., Ijaz N., Yaseen T., Iqbal R., Murtaza G., Usman M., Sampath S. (2024). Regulatory and Ethical Concerns of Nanotechnology in Agriculture.

[194] Shelar A., Nile S.H., Singh A.V., Rothenstein D., Bill J., Xiao J., Chaskar M., Kai G., Patil R. (2023). Recent Advances in Nano-Enabled Seed Treatment Strategies for Sustainable Agriculture: Challenges, Risk Assessment, and Future Perspectives. Nano-Micro Lett..

[195] Chaud M., Souto E.B., Zielinska A., Severino P., Batain F., Oliveira-Junior J., Alves T. (2021). Nanopesticides in Agriculture: Benefits and Challenge in Agricultural Productivity, Toxicological Risks to Human Health and Environment. Toxics.

[196] Prasad M., Mahawer S.K. (2023). Nano-Agrochemicals: Risk Assessment and Management Strategies. Plant Health Arch..

[197] Singh D., Gurjar B.R. (2022). Nanotechnology for Agricultural Applications: Facts, Issues, Knowledge Gaps, and Challenges in Environmental Risk Assessment. J. Environ. Manag..

[198] Wang P., Lombi E., Menzies N.W., Zhao F.-J., Kopittke P.M. (2018). Engineered Silver Nanoparticles in Terrestrial Environments: A Meta-Analysis Shows That the Overall Environmental Risk Is Small. Environ. Sci. Nano.

[199] Adisa I.O., Pullagurala V.L.R., Peralta-Videa J.R., Dimkpa C.O., Elmer W.H., Gardea-Torresdey J.L., White J.C. (2019). Recent Advances in Nano-Enabled Fertilizers and Pesticides: A Critical Review of Mechanisms of Action. Environ. Sci. Nano.

[200] Kopittke P.M., Lombi E., Wang P., Schjoerring J.K., Husted S. (2019). Nanomaterials as Fertilizers for Improving Plant Mineral Nutrition and Environmental Outcomes. Environ. Sci. Nano.

[201] Ruffatto K., Minello L.V.P., Furtado B.G., Johann L., Sperotto R.A. (2025). Nanoparticles as Tools for Enhancing Plant Resistance to Biotic Stress in the Context of Climate Change. Physiol. Plant..

[202] Damalas C.A., Eleftherohorinos I.G. (2011). Pesticide Exposure, Safety Issues, and Risk Assessment Indicators. Int. J. Environ. Res. Public Health.

[203] Singh A.V., Maharjan R.-S., Kanase A., Siewert K., Rosenkranz D., Singh R., Laux P., Luch A. (2021). Machine-Learning-Based Approach to Decode the Influence of Nanomaterial Properties on Their Interaction with Cells. ACS Appl. Mater. Interfaces.

[204] Mwaanga P. (2018). Risks, Uncertainties, and Ethics of Nanotechnology in Agriculture. New Visions in Plant Science.

